# IL-10 constrains sphingolipid metabolism to limit inflammation

**DOI:** 10.1038/s41586-024-07098-5

**Published:** 2024-02-21

**Authors:** Autumn G. York, Mathias H. Skadow, Joonseok Oh, Rihao Qu, Quan D. Zhou, Wei-Yuan Hsieh, Walter K. Mowel, J. Richard Brewer, Eleanna Kaffe, Kevin J. Williams, Yuval Kluger, Stephen T. Smale, Jason M. Crawford, Steven J. Bensinger, Richard A. Flavell

**Affiliations:** 1 Department of Immunobiology, Yale University, New Haven, CT USA; 2 Howard Hughes Medical Institute, Yale University, New Haven, CT USA; 3 Department of Chemistry, Yale University, New Haven, CT USA; 4 Institute of Biomolecular Design and Discovery, Yale University, West Haven, CT USA; 5 Computational Biology and Bioinformatics Program, Yale University, New Haven, CT USA; 6 Department of Microbiology, Immunology and Molecular Genetics, UCLA, Los Angeles, CA USA; 7 Department of Biological Chemistry, David Geffen School of Medicine, UCLA, Los Angeles, CA USA; 8 UCLA Lipidomics Laboratory, Los Angeles, CA USA; 9 Department of Microbial Pathogenesis, Yale University School of Medicine, New Haven, CT USA; 10 Present Address: Department of Immunology, School of Medicine, University of Washington, Seattle, WA USA

**Keywords:** Toll-like receptors, Sphingolipids, Lipidomics, Mucosal immunology, Chronic inflammation

## Abstract

Interleukin-10 (IL-10) is a key anti-inflammatory cytokine that can limit immune cell activation and cytokine production in innate immune cell types^1^. Loss of IL-10 signalling results in life-threatening inflammatory bowel disease in humans and mice—however, the exact mechanism by which IL-10 signalling subdues inflammation remains unclear^2–5^. Here we find that increased saturated very long chain (VLC) ceramides are critical for the heightened inflammatory gene expression that is a hallmark of IL-10 deficiency. Accordingly, genetic deletion of ceramide synthase 2 (encoded by *Cers2*), the enzyme responsible for VLC ceramide production, limited the exacerbated inflammatory gene expression programme associated with IL-10 deficiency both in vitro and in vivo. The accumulation of saturated VLC ceramides was regulated by a decrease in metabolic flux through the de novo mono-unsaturated fatty acid synthesis pathway. Restoring mono-unsaturated fatty acid availability to cells deficient in IL-10 signalling limited saturated VLC ceramide production and the associated inflammation. Mechanistically, we find that persistent inflammation mediated by VLC ceramides is largely dependent on sustained activity of REL, an immuno-modulatory transcription factor. Together, these data indicate that an IL-10-driven fatty acid desaturation programme rewires VLC ceramide accumulation and aberrant activation of REL. These studies support the idea that fatty acid homeostasis in innate immune cells serves as a key regulatory node to control pathologic inflammation and suggests that ‘metabolic correction’ of VLC homeostasis could be an important strategy to normalize dysregulated inflammation caused by the absence of IL-10.

## Abstract (web summary)

IL-10 exerts its anti-inflammatory activity in macrophages by increasing the expression of enzymes that promote fatty acid desaturation and downstream regulation of the transcription factor REL.

## Main

IL-10 is an anti-inflammatory cytokine that limits immune activation of innate and adaptive immune cells^1^. IL-10 signalling has an important role in modulating mucosal inflammation in the intestine, and deletion of the cytokine or its receptor (IL-10R) results in severe inflammatory bowel disease (IBD) in both mouse and humans^2–5^. Despite the clear importance of IL-10 in maintaining intestinal homeostasis, the exact mechanism of how IL-10–IL-10R signalling reduces inflammation is not well understood. Accumulated work has revealed that inflammatory signals rapidly rewire lipid metabolic programmes of immune cells^6–14^ to support inflammation and effector functions, leading to the hypothesis that anti-inflammatory cytokines such as IL-10 may direct changes in lipid metabolism to counteract inflammatory stimuli. Here we test this supposition and uncover a role for IL-10 signalling in the regulation of sphingolipid metabolism in macrophages downstream of toll-like receptor 2 (TLR2). In the absence of IL-10, we find that TLR2-activated macrophages have increased metabolic flux through the de novo sphingolipid biosynthesis pathway, resulting in the accumulation of endogenously synthesized ceramides. We found that increased levels of saturated VLC ceramides specifically contributed to inflammatory phenotypes both in vitro and in vivo. Surprisingly, altered sphingolipid metabolism in IL-10 deficient cells was mediated by reduced synthesis of mono-unsaturated fatty acids (MUFAs), and could be corrected by providing exogenous MUFAs. We find that the prolonged inflammatory gene expression programme enforced by altered VLC ceramide homeostasis requires the NF-κB family transcription factor REL. These studies provide strong evidence that coordinate regulation of lipid metabolism by IL-10 is necessary to constrain REL-dependent pathologic inflammation and suggest that targeting specific aspects of lipid homeostasis in the intestine could control aberrant inflammation underlying IBD.

## IL-10 regulates sphingolipid metabolism

**Fig. 1:**
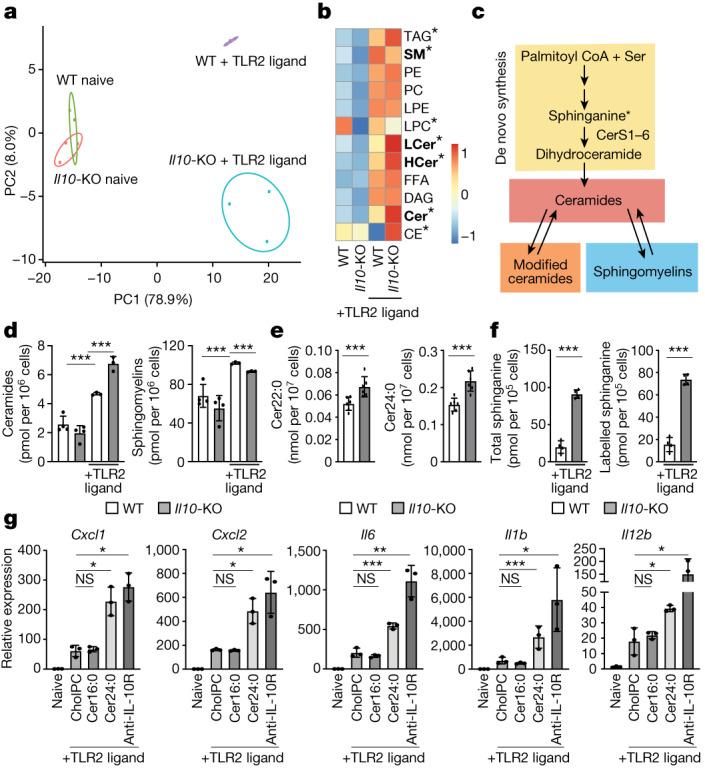
IL-10 signalling regulates sphingolipid metabolism. **a**, Principal component analysis (PCA) of individual lipids quantified by mass spectrometry from naive or TLR2-activated (50 ng ml^−1^ Pam3CysK4) wild-type and *Il10*-KO BMDMs for 48 h. The percentage of total variance explained by individual principal components (PC1 and PC2) is indicated. Prediction ellipses are set at 95% probability (*n* = 3–4). **b**, Heat map of individual lipid species measured by direct infusion mass spectrometry from naive BMDMs (left two columns) or TLR2-activated BMDMs (right two columns) stimulated as in **a**. Scaled by row (lipid species). Bolded text indicates *P < 0.05 between TLR2-activated wild-type and *Il10*-KO BMDMs. CE, cholesteryl esters; Cer, ceramides; DAG, diacylglycerols; FFA, free fatty acids; HCer, hexosyl ceramides; LCer, lactosyl ceramides; LPC, lysophosphatidylcholine; LPE, lysophosphatidylethanolamine; PC, phosphatidylcholine; PE, phosphatidylethanolamine; SM, sphingomyelins; TAG, triglycerides. **c**, Simplified schematic of sphingolipid metabolism. Ceramides are generated by de novo synthesis pathway at the endoplasmic reticulum. Ceramides can be further modified to generate hexosyl and lactosyl ceramides (modified ceramides), which can be broken back down into ceramides. Ceramides serve as the building blocks for all sphingomyelin species, which can also be broken down into ceramides. **d**, Total ceramides and sphingomyelin species measured by direct infusion mass spectrometry from BMDMs stimulated as in **a** (*n* = 3–4). **e**, Ceramide species measured by direct infusion mass spectrometry from ex vivo peritoneal macrophages collected from wild-type and *Il10*-KO mice after 48 h TLR2 ligand (50 μg Pam3CysK4 per mouse) administered via intraperitoneal injection (*n* = 6). **f**, LC–MS analysis of total and labelled sphinganine in 48 h TLR2-activated wild-type and *Il10*-KO BMDMs. (*n* = 4). **g**, Quantitative PCR (qPCR) analysis of inflammatory gene expression in naive BMDMs or BMDMs activated with TLR2 ligand (50 ng ml^−1^ Pam3CysK4) for 24 h. TLR2-activated macrophages were incubated with cholesteryl:phosphatidylcholine (cholPC) alone or with cholPC loaded with Cer16:0 or Cer24:0 or cholPC plus neutralizing anti-IL-10R (5 μg ml^−1^) for the last 20 h of the activation (*n* = 3 for each group). All lipids administered at a final concentration of 30 μM. All data are mean of biological replicates ± s.d. *P < 0.05, **P < 0.01, ***P < 0.005 (two-tailed unpaired Student’s *t*-test). Source Data

**Extended Data Fig. 1:**
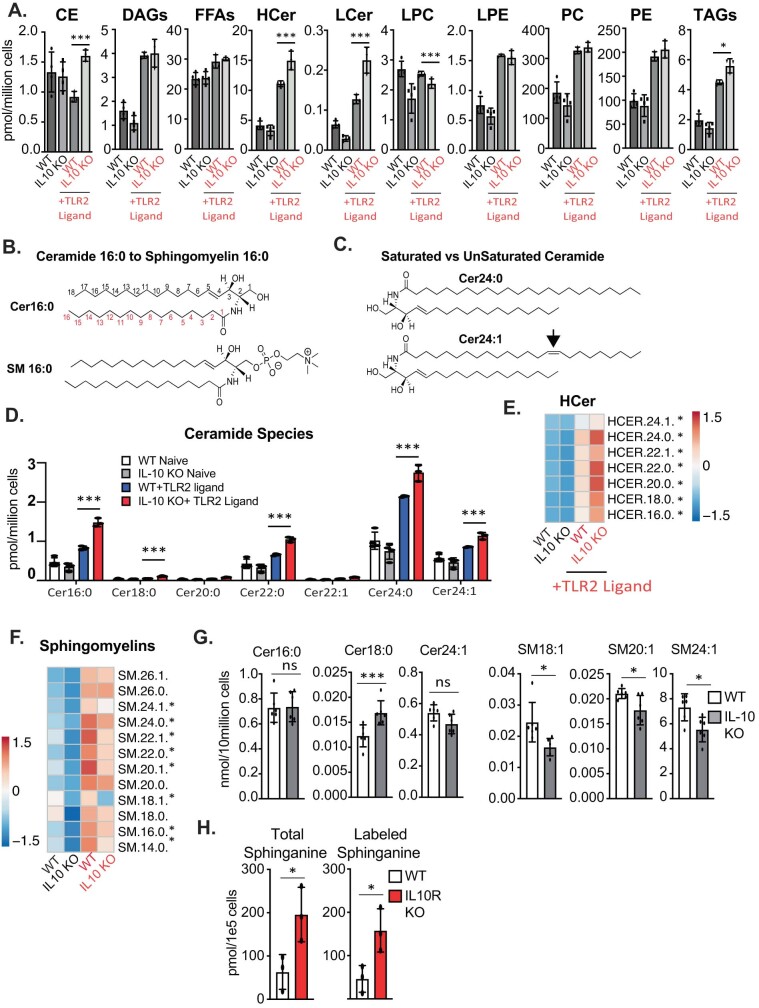
IL-10 signaling controls sphingolipid metabolism. A) Total Cholesteryl Esters (CE), Diacylglycerols (DAG), Free Fatty Acids (FFAs), Hexosyl Ceramides (HCer), Lactosyl Ceramides (LCer), Lysophosphatidylcholine (LPCs), Lysophosphatidylethanolamine (LPE), phosphatidylcholine (PC), phosphatidylethanolamine (PE) and triglycerides (TAGs) measured by direct infusion MS from naïve or 48 h TLR2 activated WT and IL10 KO BMDMs (n = 3-4). B) Schematic of Ceramide 16:0 and Sphingomyelin (SM) 16:0. Both sphingolipids contain an 18 carbon long constant chain (top, black numbers), and a 16 carbon long variable chain (bottom, red numbers). Figure made with ChemDraw V22.2.0.3300. C) Schematic of saturated (Cer24:0) versus unsaturated (Cer24:1) ceramides. Double bond in 24:1 is located at the arrow. Figure made with ChemDraw V22.2.0.3300. D) Ceramide species measured by direct infusion MS from WT and IL10 KO BMDMs that are naïve or activated with TLR2 ligand (50 ng/mL Pam3CysK4) for 48 h. E) Heat map of individual hexosyl ceramide (HCer) species measured by direct infusion MS from WT and IL10 KO BMDMs that are naïve or activated with TLR2 ligand (50 ng/mL Pam3CysK4) for 48 h. Scaled by row (lipid species) * indicate significance of p < 0.05 between WT and IL-10 KO activated with TLR2. F) Heat map of individual sphingomyelin (SM) species measured by direct infusion MS from WT and IL10 KO BMDMs that are naïve or activated with TLR2 ligand (50 ng/mL Pam3CysK4) for 48 h. Scaled by row (lipid species) * indicate significance of p < 0.05 between WT and IL-10 KO activated with TLR2. G) Total Ceramide and Sphingomyelins (SM) measured by direct infusion MS from ex vivo peritoneal macrophage collected from WT and IL10 KO mice after 48 h TLR2 ligand (50ug/mouse Pam3CysK4) administered via IP injection (n = 6). H) LC-MS analysis of total and labeled sphinganine in 48 h TLR2-activated WT and IL-10R KO BMDMs (n = 3). All experiments are reported as means ± SD from 3 independent experiments, unless noted otherwise. *P < 0.05; **P < 0.01, ***P < 0.005 (two-tailed unpaired Student’s *t* test). Source Data

Previous work has shown that inflammatory TLR signals profoundly reshape the lipid composition of macrophages to influence effector function^10^. IL-10 is produced downstream of select TLRs to promote the resolution of inflammation, leading us to explore whether IL-10 signalling was required for lipid metabolic reprogramming downstream of TLR2 activation. To that end, we compared lipid profiles of naive or TLR2-activated wild-type and *Il10*-knockout (KO) bone marrow-derived macrophages (BMDMs) using targeted shotgun lipidomic analysis. We detected approximately 1,100 lipid species from 13 lipid subclasses. Principal component analysis of lipidomics data showed that naive wild-type and *Il10*-KO macrophages were largely similar for principal components PC1 and PC2 (Fig. 1a). PC1 captured approximately 80% of the variance observed in TLR2-activated macrophages in wild-type and *Il10*-KO macrophages. PC2 (8% of variance) delineated the influence of IL-10 signalling of lipid composition downstream of TLR2 (Fig. 1a). Closer inspection of lipids influenced specifically by IL-10 signalling after TLR2 activation revealed a marked accumulation of ceramides, modified ceramides (hexosyl ceramides and lactosyl ceramides), and a decrease in sphingomyelins (Fig. 1b–d and Extended Data Fig. 1a).

Ceramides and sphingomyelins contain a sphingoid base and an N-acylated fatty acyl chain of variable length^15^ (Extended Data Fig. 1b). Mammalian cells generate ceramides with acyl tail lengths of 14–18 carbons (long chain sphingolipids) or 20 or more carbons (VLC sphingolipids) that can be saturated (no double bonds) or mono-unsaturated (one double bond) (Extended Data Fig. 1c). Using our methodology, we were able to consistently quantify sphingolipid species with saturated and mono-unsaturated acyl tails from 16–24 carbons in macrophage samples. Compared with wild-type counterparts, all species of ceramides and all hexosyl ceramides were increased in activated *Il10*-KO macrophages (Extended Data Fig. 1d,e). By contrast, all unsaturated sphingomyelins and several saturated sphingomyelins were decreased in the *Il10*-KO macrophages (Extended Data Fig. 1f).

To test if this observation was true in vivo, we preformed lipdiomic analysis on ex vivo peritoneal macrophages collected from wild-type or* Il10*-KO mice that received TLR2 agonists via intraperitoneal injection. In line with our in vitro results, we find that IL-10 deficient ex vivo macrophages exhibit significant increases in many ceramide species (Fig. 1e and Extended Data Fig. 1g) and significant decreases in mono-unsaturated sphingomyelin species (Extended Data Fig. 1g). Thus, we conclude that IL-10 signalling regulates sphingolipid metabolism of macrophages in response to inflammatory stimuli both in vitro and in vivo.

Ceramides can be generated via a de novo synthesis pathway and can be further modified to generate complex sphingolipids such as sphingomyelins, and lactosyl- or hexosyl-modified ceramides. Alternatively, salvage pathways can convert downstream products back to ceramides. (Fig. 1c) (reviewed in ref. ^16^). Several studies have found that inflammation can affect sphingolipid salvage pathways^17,18^, but less is known about how inflammatory signals affect the regulation of de novo sphingolipid synthesis. To understand whether IL-10 signalling influences ceramide synthesis, we analysed flux into the de novo sphingolipid synthesis pathway using stable-isotope tracers. Macrophage cultures were fed U-^13^C,^15^N-serine (U denotes universally labelled), a required metabolite for the first step of sphingolipid synthesis (Fig. 1c). Targeted liquid chromatography mass spectrometry (LC–MS) of macrophages deficient in IL-10 signalling (*Il10-KO* or *Il10rb*-KO) contained more total and isotope-labelled sphinganine compared with wild-type controls (Fig. 1f and Extended Data Fig. 1h), indicating that increased de novo ceramide synthesis contributes to the aberrant accumulation of ceramides observed in macrophages that lack IL-10 signalling.

**Extended Data Fig. 2:**
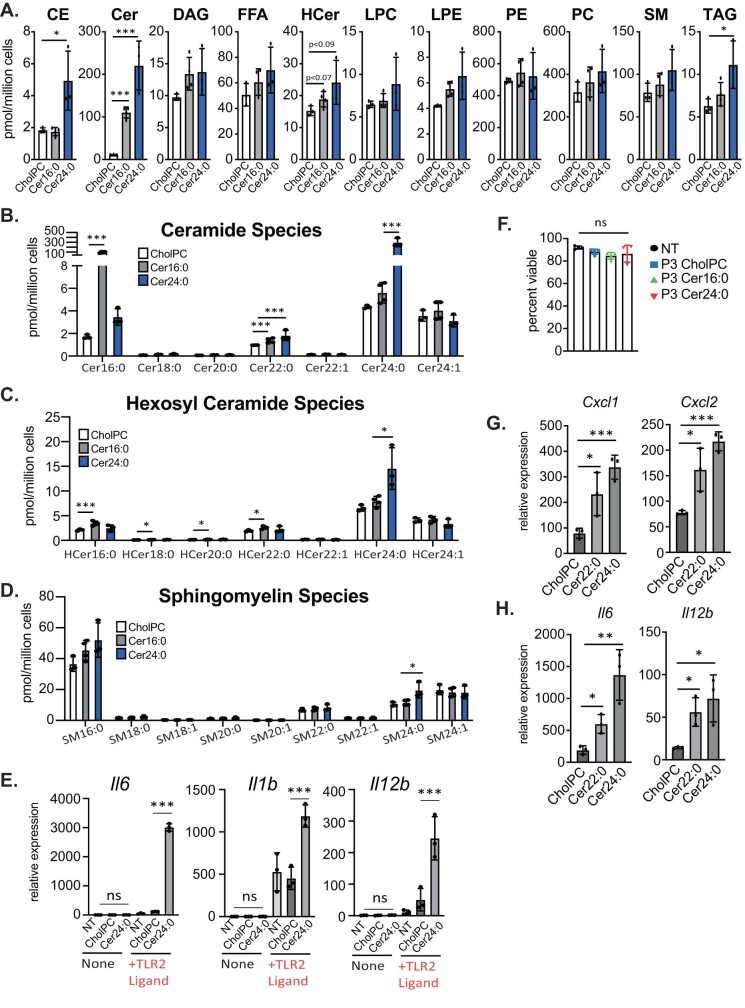
Exogenous very long chain ceramides induce inflammation. A) Total Cholesteryl Esters (CE), Ceramides (Cer), Diacylglycerols (DAG), Free Fatty Acids (FFAs),Hexosyl Ceramides (HCer), Lysophosphatidylcholine (LPCs), Lysophosphatidylethanolamine (LPE), phosphatidylcholine (PC), phosphatidylethanolamine (PE) and triglycerides (TAGs) measured by direct infusion MS from naïve or 48 h TLR2 activated WT BMDMs treated with CholPC (vehicle), Cer16:0 or Cer24:0. All lipids are administered at final concentration of 30uM. B) Ceramide species measured by direct infusion MS from naïve or 48 h TLR2 activated WT BMDMs treated with CholPC (vehicle), Cer16:0 or Cer24:0. All lipids are administered at final concentration of 30uM. C) Hexosyl Ceramide species measured by direct infusion MS from naïve or 48 h TLR2 activated WT BMDMs treated with CholPC (vehicle), Cer16:0 or Cer24:0. All lipids are administered at final concentration of 30uM. D) Sphingomyelin species measured by direct infusion MS from naïve or 48 h TLR2 activated WT BMDMs treated with CholPC (vehicle), Cer16:0 or Cer24:0. All lipids are administered at final concentration of 30uM. E) qPCR analysis of *Il6, Il1b* and *Il12b* gene expression in naïve BMDMs treated with CholPC or Cer24:0 alone for 48 h versus TLR2-activated BMDMs treated with the same lipids (30uM for all treatments) (n = 3). F) Percent viability measured by AOPI staining for naïve or TLR2- activated BMDMs treated with CholPC, Cer16:0 or Cer24:0 (30uM for all lipids) for 48 h. G) qPCR analysis of *Cxcl1* and *Cxcl2* gene expression in BMDMs activated with TLR2 ligand (50 ng/mL Pam3CysK4) for 48 h. TLR2-activated macrophage were incubated with Cholesteryl:Phosphatidylcholine (CholPC) lipid sheets alone or with CholPC loaded with ceramide 22:0 (Cer22:0) or ceramide 24:0 (Cer24:0) for the last 44 h of the activation (n = 3 for each group). All lipids administered at a final concentration of 30uM. H) qPCR analysis of *Il6* and *Il12b* gene expression in BMDMs activated with TLR2 ligand (50 ng/mL Pam3CysK4) for 48 h. TLR2-activated macrophage were incubated with Cholesteryl:Phosphatidylcholine (CholPC) lipid sheets alone or with CholPC loaded with ceramide 22:0 (Cer22:0) or ceramide 24:0 (Cer24:0) for the last 44 h of the activation (n = 3 for each group). All lipids administered at a final concentration of 30uM. All experiments are reported as means ± SD from 3 independent experiments, unless noted otherwise. *P < 0.05; **P < 0.01, ***P < 0.005 (two-tailed unpaired Student’s *t* test). Source Data

We next tested whether accumulation of ceramides contributes to inflammatory gene expression programme of TLR2-activated macrophages. To do so, we generated lipid bilayers consisting of cholesteryl-phosphocholine that were either left empty (denoted CholPC)  or complexed with ceramide 16:0 (Cer16:0, the most abundant long chain ceramide) or ceramide 24:0 (Cer24:0, the most abundant VLC ceramide) (Extended Data Fig. 1d). As expected, exogenous ceramides increased downstream sphingolipids metabolites without largely altering other lipid classes (Extended Data Fig. 2a–d). In naive macrophages, cholPC or ceramides alone did not affect inflammatory gene expression (Extended Data Fig. 2e). Similarly, the addition of Cer16:0 to TLR2-activated wild-type macrophages did not alter inflammatory gene expression. By contrast, addition of Cer24:0 upregulated inflammatory gene expression to levels similar to those seen in the absence of IL-10/IL-10R signalling (Fig. 1g), without affecting macrophage viability (Extended Data Fig. 2f). Addition of Cer22:0, the second most abundant VLC ceramide (Extended Data Fig. 1d), also induced inflammatory gene expression similar to that resulting from Cer24:0 addition (Extended Data Fig. 2g,h). Together, these data indicate that accumulation of VLC ceramides can enhance cytokine and chemokine gene expression in TLR-activated macrophages, and suggests that dysregulation of VLC ceramide homeostasis contributes to the exacerbated inflammation observed in IL-10 deficiency.

## Loss of VLC ceramides limits inflammation

**Extended Data Fig. 3:**
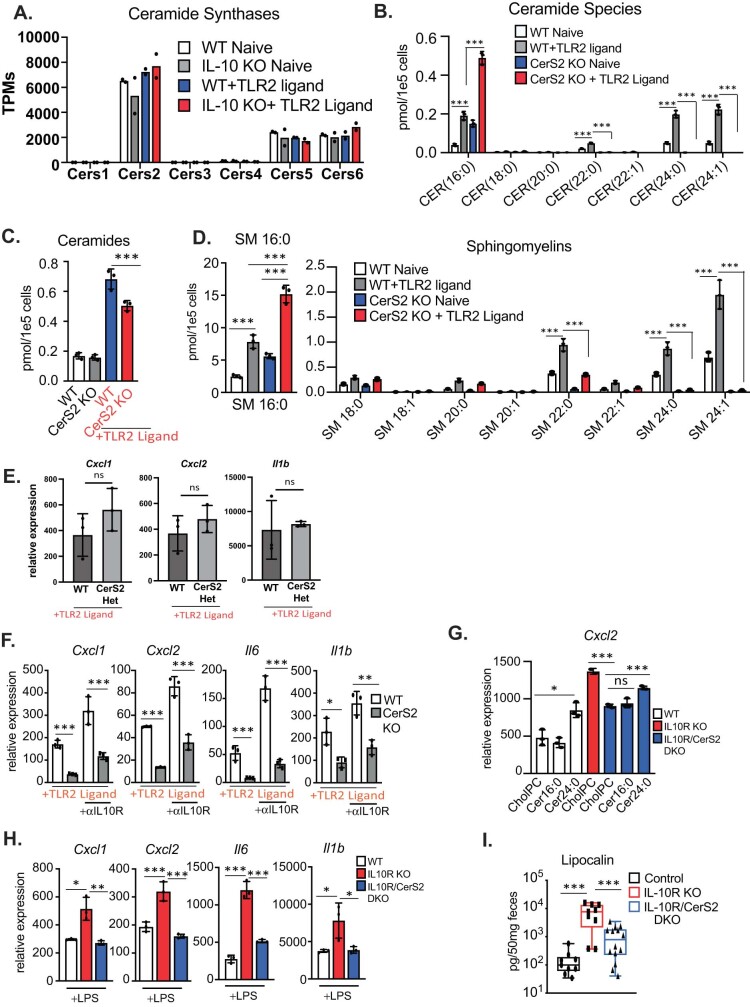
CerS2 is required for VLC ceramide production and inflammation. A) RNAseq analysis of Ceramide synthase genes from naïve or TLR2 activated WT and IL10 KO BMDMs from RNAseq analysis from samples matched to lipidomics analysis in Fig. 1 (TPMs must be above 0 to be listed). (n = 2). B) Ceramide species measured by direct infusion MS from naïve or 48 h TLR2-activated WT or CerS2 KO BMDMs (n = 3). C) Total ceramides measured by direct infusion MS from naïve or 48 h TLR2-activated WT or CerS2 KO BMDMs (n = 3 per group). D) Sphingomyelin (SM) species measured by direct infusion MS from naïve or 48 h TLR2-activated WT or CerS2 KO BMDMs (n = 3). E) qPCR analysis of *Cxcl1, Cxcl2*, and *Il1b* gene expression in 48 h TLR2-activated (50 ng/mL Pam3CysK4) WT or CerS2 Heterzygous BMDMs (n = 3 per group). F) qPCR analysis of *Cxcl1, Cxcl2, Il6*, and *I1b* gene expression in 48 h TLR2-activated (50 ng/mL Pam3CysK4) WT or CerS2 KO BMDMs +/− anti-IL-10R neutralizing antibody (5ug/mL) for the last 44 h of the activation (n = 3 per group). G) qPCR analysis of *Cxcl2* gene expression in 48 h TLR2-activated (50 ng/mL Pam3CysK4) WT (white bars), IL-10R KO (red bar) or IL-10R/CerS2 DKO (Blue bars) peritoneal macrophage supplemented with CholPC (vehicle), Cer16:0 or Cer24:0 for the last 44 h of activation. H) qPCR analysis of *Cxcl1, Cxcl2, Il6*, and *Il1b* gene expression in 48 h TLR4-activated (50 ng/mL LPS) WT (white bars), IL-10R KO (red bar) or IL-10R/CerS2 DKO (Blue bars) peritoneal macrophage for 48 h. I) ELISA analysis of lipocalin from feces of control (IL-10R Heterozygous), IL-10R KO and IL-10R/CerS2 DKO chimeric mice (n = 9−14) Statistical differences measured by two-sided Mann-Whitney tests. All experiments are reported as means ± SD from 3 independent experiments, unless noted otherwise. *P < 0.05; **P < 0.01, ***P < 0.005 (two-tailed unpaired Student’s *t* test). Source Data

The length of the variable acyl tail (Extended Data Fig. 1b) incorporated into ceramides is specified during de novo synthesis by ceramide synthases (CerS) (Fig. 1c). In mouse and humans, there are six ceramide synthases (CerS1–6 (also known as Lass1–6)). RNA-sequencing (RNA-seq) data from naive and TLR2-activated wild-type and *Il10*-KO macrophages (matched to the lipidomics dataset; cells were prepared and stimulated at the same time as cells from the lipidomics experiments in Fig. 1 but were collected 24 h after TLR2 activation) showed that BMDMs highly express *Cers2*, *Cers5* and *Cers6* (Extended Data Fig. 3a). In non-immune cell types, CerS2 is reported to regulate the synthesis of VLC ceramides Cer20:0–Cer26:0^19^. To determine whether CerS2 regulates VLC ceramides in macrophages, we generated *Cers2-*KO mice and performed lipidomics on BMDMs from knockout and control mice. Consistent with studies in non-immune cells, *Cers2-*KO BMDMs have nearly undetectable levels of ceramides with acyl chains longer than 20 carbons (Extended Data Fig. 3b), resulting in a net decrease in total ceramides (Extended Data Fig. 3c). Accordingly, loss of CerS2 markedly reduced the amount of VLC sphingomyelin species (Extended Data Fig. 3d). Thus, loss of CerS2 in TLR2-activated macrophages disrupts VLC ceramide and sphingomyelin homeostasis.

**Fig. 2:**
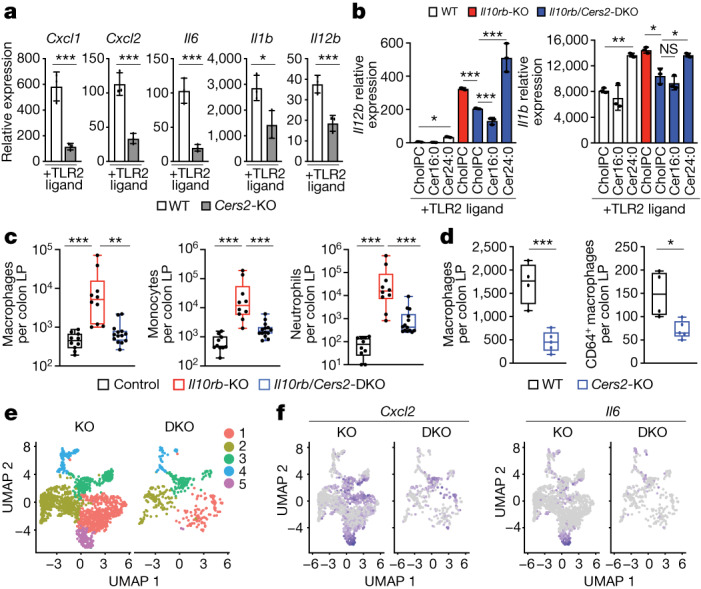
Genetic inhibition of VLC ceramide synthesis limits inflammation. **a**, qPCR analysis of Inflammatory gene expression in 48 h TLR2-activated (50 ng ml^−1^ Pam3CysK4) wild-type or *Cers2-*KO BMDMs (*n* = 3 per group). **b**, qPCR analysis of *Il12b* and *Il1b* gene expression in 48 h TLR2-activated (50 ng ml^−1^ Pam3CysK4) wild-type, *Il10rb*-KO or *Il10rb*/*Cers2*-DKO peritoneal macrophages supplemented with cholPC (vehicle), Cer16:0 or Cer24:0 for the last 44 h of activation (*n* = 3 per group). All lipids administered at a final concentration of 30 μM. **c**, Macrophages (CD11c^+^MHCII^+^), monocytes (CD11b^+^Ly6C^+^) and neutrophils (Cd11b^+^Ly6G^+^) from flow cytometry-based immune cell profiling of the colonic lamina propria (LP) from control (*Il10rb*-heterozygous), *Il10rb*-KO and *Il10rb*/*Cers2*-DKO chimeric mice (*n* = 10–13). Mann–Whitney *t*-test. **d**, Total macrophages (CD11c^+^MHCII^+^) and CD64^+^ macrophages from colonic lamina propria from wild-type and *Cers2-*KO chimeric mice (*n* = 4–5). **e**, UMAP analysis of single-cell RNA-seq (scRNA-seq) data from *Il10rb*-KO and *Il10rb*/*Cers2*-DKO macrophage clusters in cells sorted from the colon lamina propria. **f**, UMAP analysis of *Cxcl2* and *Il6* scRNA-seq data from sorted macrophages from the colon lamina propria of *Il10rb*-KO or *Il10rb*/*Cers2*-DKO chimeric mice. All experiments are reported as mean of biological replicates ± s.d. *P < 0.05, **P < 0.01, ***P < 0.005 (two-tailed unpaired Student’s *t*-test, unless noted otherwise). Source Data

To determine whether CerS2 and its sphingolipid products are important for inflammation, we generated BMDMs from wild-type and *Cers2-*KO mice and activated them with TLR2 ligand. Whereas *Cers2*-heterozygous macrophages exhibited similar inflammatory gene expression to wild-type counterparts (Extended Data Fig. 3e), *Cers2-*KO macrophages showed significantly reduced inflammatory gene expression in response to TLR2 ligands (Fig. 2a) and did not fully induce inflammatory gene expression during IL-10R blockade (Extended Data Fig. 3f). This suggests that CerS2 function is important for TLR2-driven macrophage inflammation alone or in the context of IL-10 deficiency. In line with this idea, loss of CerS2 on the IL-10R-deficient background significantly reduced macrophage inflammatory gene expression in response to TLR2 (Fig. 2b and Extended Data Fig. 3g) and TLR4 ligands (Extended Data Fig. 3h). Notably, addition of exogenous Cer24:0, but not Cer16:0, restored inflammation in the *Il10rb*/*Cers2*-DKO macrophages (Fig. 2b and Extended Data Fig. 3g), indicating that accumulation of VLC ceramides, the lipid products of CerS2, are important for mediating the increased inflammatory gene expression in IL-10-deficient macrophages.

**Extended Data Fig. 4:**
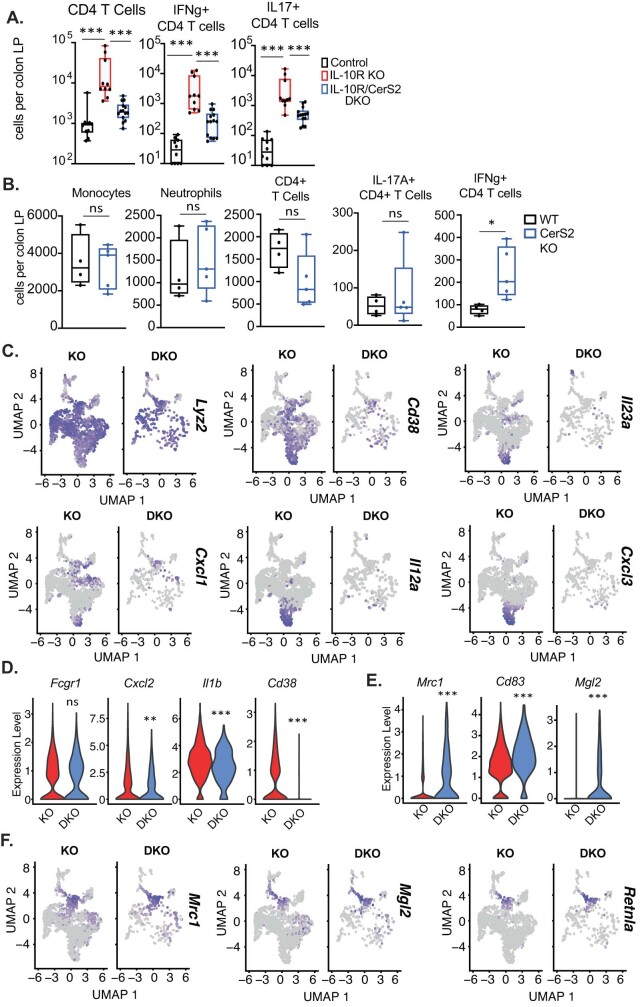
CerS2 regulates colonic inflammation in vivo. A) Flow cytometry-based immune cell profiling of the total CD4 T cells (Lin-/TCRB +/CD4 +) or IFNg+ or IL-17A + CD4 T cells from colonic lamina propria from control (IL-10R Heterozygous), IL-10R KO and IL-10R/CerS2 DKO chimeric mice. B) Flow cytometry-based immune cell profiling of the colonic lamina propria from WT and CerS2 KO chimera mice. C) scRNAseq UMAP analysis of macrophage markers and inflammatory genes from macrophage from IL-10R KO and IL-10R/CerS2 DKO colon LPs. D) scRNAseq gene expression analysis from all clusters of *Fcgr1* (CD64), *Cxcl2*, *Il1b* and *Cd38* from sorted macrophage from the colon LP of IL-10R KO or IL-10R/CerS2 DKO chimera mice. E) scRNAseq gene expression analysis from all clusters of *Mrc1, CD83* and *Mgl2* from sorted macrophage from the colon LP of IL-10R KO or IL-10R/CerS2 DKO chimera mice. F) scRNAseq UMAP analysis of macrophage tolerogenic and M2 markers as in S5E from macrophage from IL-10R KO and IL-10R/CerS2 DKO colon LPs. All experiments are reported as means ± SD from 3 independent experiments, unless noted otherwise. *P < 0.05; **P < 0.01, ***P < 0.005 (two-tailed unpaired Student’s *t* test). Source Data

Since loss of CerS2 reduced inflammatory gene expression in IL-10R-deficient macrophages, we hypothesized that CerS2 may be important for regulating the colonic inflammation that is the hallmark of *Il10*-KO or *Il10rb*-KO mice^2–4^. We observed that *Cers2*-KO and *Cers2*- and *Il10rb*-double knockout (DKO) mice were small in stature, had spontaneous seizures, and often died before 10 weeks of age in our colony. This is consistent with phenotypes published by other groups that show that in vivo *Cers2* deletion results in defects in myelin sheath production and spontaneous seizures^20^. To circumvent complications resulting from these systemic issues, we generated bone marrow chimeras from CD45.2^+^ *Il10rb*^+/−^ (control) mice, *Il10rb*-KO mice and *Il10rb*/*Cers2*-DKO littermate mice into CD45.1^+^ recipients. Microbial variation was minimized by cohousing chimeric mice in a 2 × 2 × 2 format for each donor genotype. Bedding was also transferred equally between cages every other week. At 10 weeks after engraftment, faecal samples from the chimeric mice were collected to quantify lipocalin, a canonical marker of colonic inflammation^21^. At this timepoint, we observed no overt signs of colonic inflammation (for example, rectal prolapse or weight loss; data not shown). However, consistent with previous studies in whole-body *Il10*-KO and *Il10rb-*KO mice^21^, *Il10rb*-KO chimeric mice had significantly higher faecal lipocalin compared with control mice (Extended Data Fig. 3i). Notably, *Il10rb*/*Cers2*-DKO chimeric mice had reduced faecal lipocalin compared with IL-10R KO chimeric mice in both male and female mice (Extended Data Fig. 3i and data not shown). To further examine the importance of CerS2 in vivo, we collected colon tissue from the chimeric mice, isolated the immune cells from the colon lamina propria and assessed cell populations by flow cytometry (for gating strategies, see Supplementary Fig. 1). Consistent with previous studies^4^, *Il10rb*-KO chimeras had increased inflammatory cell infiltrates, including macrophages, monocytes, neutrophils, total CD4^+^ T cells, T_H_1 (IFNγ^+^) and T_H_17 (IL-17A^+^) cells (Fig. 2c and Extended Data Fig. 4a). Markedly, loss of CerS2 on the IL-10R-deficient background reduced immune cell infiltrates compared with the *Il10rb*-KO chimeras (Fig. 2c and Extended Data Fig. 4a), indicating that synthesis of VLC ceramides is important for driving immune cell-mediated colitis in the absence of IL-10 signalling.

To further understand how CerS2 affects colonic homeostasis, we generated wild-type and *Cers2* single-knockout chimeric mice. These chimeric mice were cohoused for 12 weeks and then immune cell populations in the colonic lamina propria were analysed. In this model, there are no overt drivers of inflammation (such as loss of IL-10R), which enables us to determine whether CerS2 is important for normal immune cell homeostasis in the colon. Notably, we found no reduction in monocytes, neutrophils, total CD4 T cells, T_H_1 CD4 T cells or T_H_17 CD4 T cells in the colon lamina propria of *Cers2-*KO chimeric mice compared with wild-type controls (Extended Data Fig. 4b). However, *Cers2-*KO chimeric mice exhibited a reduction in macrophages (CD11c^+^MHCII^+^) and mature macrophages (CD11c^+^MHCII^+^CD64^+^) (Fig. 2d), suggesting that under basal conditions, CerS2 is important for maintenance of colonic macrophage populations. Colonic macrophages are continually replenished in the ‘macrophage waterfall’^22,23^, in which circulating monocytes migrate to the colon, acquire CD11c and MHCII and eventually CD64 (encoded by *Fcgr1*), and become more responsive to TLR signalling, before becoming ‘tolerogenic’ to maintain homeostasis in the colon. As macrophages are turned over, new macrophages mature to repopulate the colon. To determine how loss of CerS2 affected macrophage homeostasis in vivo, we sorted CD45^+^ immune cells from *IIl10rb*-KO or *Il10rb*/*Cers2*-DKO chimeric mice and single-cell transcriptomics performed on macrophages. Uniform manifold approximation and projection (UMAP) analysis revealed that colonic macrophages isolated from *Il10rb*-KO chimeras grouped into five distinct clusters, with cluster 5 containing highly inflammatory cells (Fig. 2e,f and Extended Data Fig. 4c). Notably, *Il10rb*/*Cers2*-DKO chimeras lacked cluster 5 and had reduced expression of inflammatory macrophage markers, cytokines and chemokines compared with *Il10rb*-KO chimeras (Fig. 2e,f and Extended Data Fig. 4c,d). Notably, CerS2-deficient macrophages showed high expression of CD206 (encoded by *Mrc1*) and other tolerogenic markers, suggesting that loss of CerS2 may promote a more tolerogenic macrophage phenotype^24–28^ compared with loss of IL-10R alone (Extended Data Fig. 4e,f). Together, these data indicate that loss of CerS2 on an IL-10R deficient background can mitigate immune cell infiltrates into the colon and reduce in vivo macrophage inflammatory gene expression compared with loss of IL-10R alone.

## MUFA synthesis limits ceramide production

**Fig. 3:**
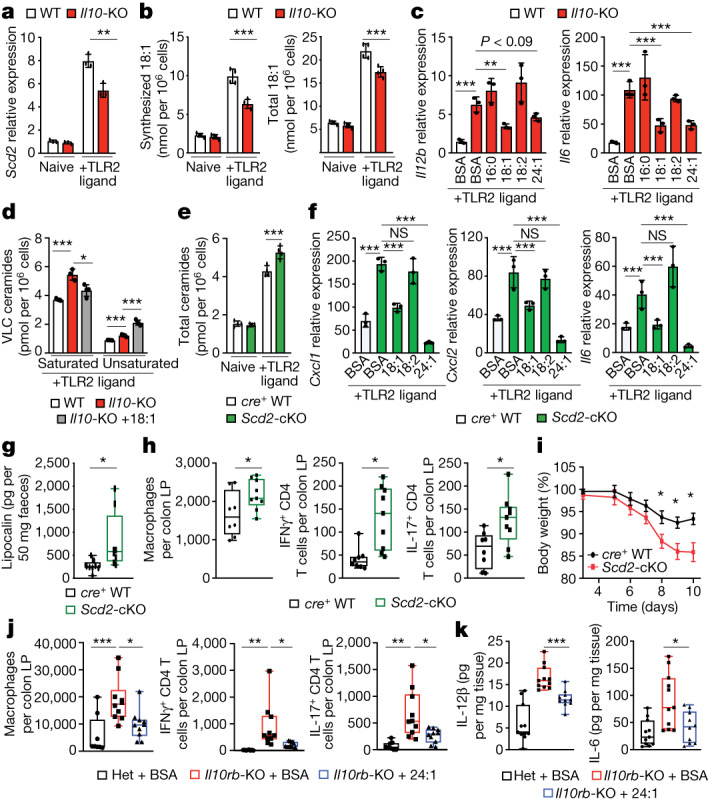
IL-10-induced mono-unsaturated fatty acid synthesis constrains ceramide production. **a**, qPCR analysis of *Scd2* gene expression in naive or 24 h TLR2-activated wild-type or *Il10*-KO BMDMs (*n* = 3). **b**, Synthesized and total oleic acid (18:1) from naive or 48 h TLR2-activated wild-type or *Il10*-KO BMDMs (*n* = 4). **c**, qPCR analysis of *Il12b* and *Il6* gene expression in 48 h TLR2-activated wild-type or *Il10*-KO BMDMs incubated with BSA, or 25 μM BSA–16:0, BSA–18:1, BSA–18:2 or BSA–24:1 for the last 44 h (*n* = 3). **d**, Saturated and unsaturated ceramide species from 48 h TLR2-activated wild-type or *Il10*-KO or *Il10*-KO BMDMs treated with 25 μM 18:1 for the last 44 h (*n* = 3–4). **e**, Total ceramides species in naive or TLR2-activated LysM-*cre*^*+*^ wild-type and *Scd2*-cKO BMDMs (*n* = 4). **f**, qPCR analysis of *Cxcl1*, *Cxcl2* and *Il6* gene expression in 48 h TLR2-activated *cre*^*+*^ wild-type BMDMs incubated with BSA, *Scd2*-cKO BMDMs incubated with BSA, and *Scd2*-cKO BMDMs incubated with 25 μM BSA–18:1, BSA–18:2 or BSA–24:1 for the last 44 h (*n* = 3). **g**, Enzyme-linked immunosorbent assay (ELISA) analysis of faecal lipocalin of control and *Scd2*-cKO male mice (*n* = 8–9). **h**, Flow cytometry-based immune cell profiling of the colonic lamina propria from naive control and *Scd2*-cKO male mice (*n* = 8–9). **i**, DSS-induced weight loss in control and *Scd2*-cKO female mice (*n* = 9). Data are mean ± s.e.m. **j**, Flow cytometry-based immune cell profiling of the colonic lamina propria from *Il10rb*-heterozygous (Het) male mice gavaged with BSA and *Il10rb*-KO male mice gavaged with BSA or BSA–24:1 (*n* = 8–10). **k**, ELISA analysis of IL-6 and IL-12β in colonic explants from *Il10rb*-heterozygous mice gavaged with BSA and *Il10rb*-KO male mice gavaged with BSA or BSA–24:1, incubated ex vivo for 24 h.(*n* = 10–11). All experiments are reported as mean of biological replicates ±  s.d. unless noted otherwise. *P < 0.05, **P < 0.01, ***P < 0.005 (two-tailed unpaired Student’s *t*-test). Source Data

**Extended Data Fig. 5:**
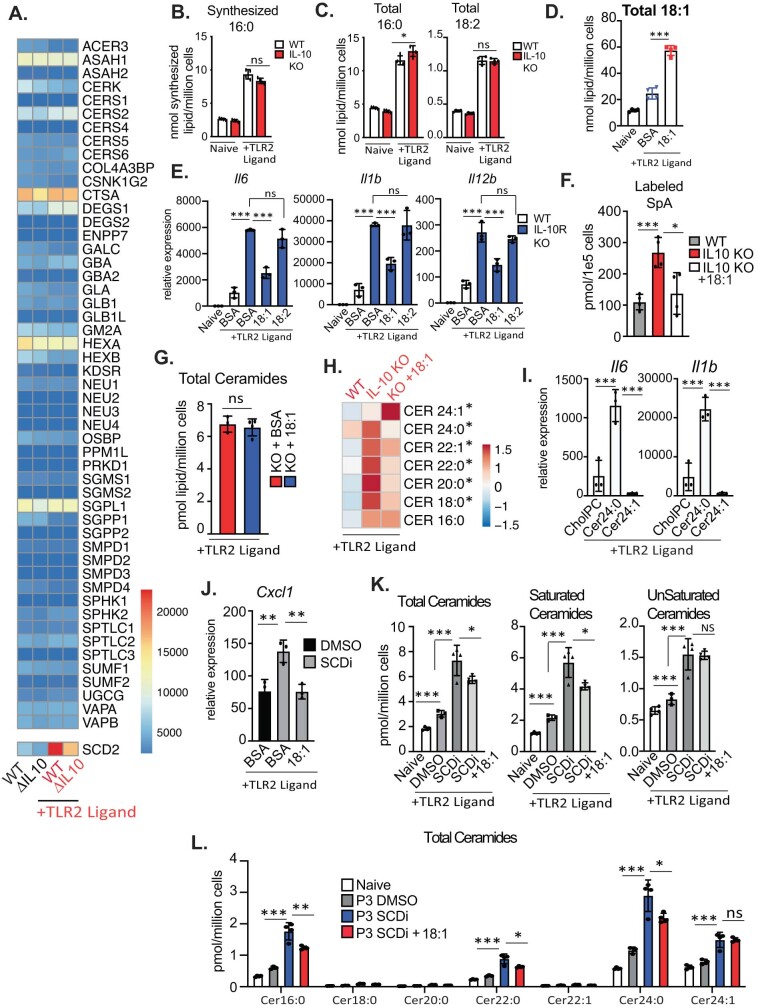
IL-10 controls MUFA synthesis. A) Heatmap of all sphingolipid metabolism pathway genes and *Scd2* from naïve or TLR2 activated WT and IL10 KO BMDMs from RNAseq analysis from samples matched to lipidomics analysis in Fig. 1 (TPMs must be above 0 to be listed). Values not scaled. B) Net synthesis (nmol/million cells) of palmitic acid (16:0) as measured by metabolic flux isotope tracer analysis in naïve or 48 h TLR2-activated (50 ng/mL Pam3CSK4) WT (white bars) or IL-10R KO (red bars) BMDMs (n = 4 per group). C) Total cellular 16:0 and 18:2 as measured by GCMS analysis of naïve or 48 h TLR2-activated (50 ng/mL Pam3CSK4) IL-10 KO BMDMs + BSA or 25uM 18:1(n = 4 per group). D) Total 18:1 in measured by GCMS analysis of naïve or 48 h TLR2-activated (50 ng/mL Pam3CSK4) IL-10 KO BMDMs + BSA or 25uM 18:1(n = 4 per group). E) qPCR analysis of *Il6, Il1b* and *Il12b* gene expression in naïve or 48 h TLR2-activated (50 ng/mL Pam3CSK4) WT (white bars) or IL-10R KO (blue bars) BMDMs incubated with vehicle (BSA), or BSA-conjugated 18:1 or 18:2 for the last 44 h of activation. (n = 3 per group). All lipids administered at a final concentration of 25 uM. F) Labeled Sphinganine measured by MS from 48 h TLR2-activated WT + BSA (vehicle), IL-10 KO + BSA (vehicle) or IL-10 KO + 25 uM 18:1 BMDMs (n = 4 per group). G) Total ceramides species measured by direct infusion MS from 48 h TLR2- activated IL-10 KO BMDMs plus BSA or BSA-18:1 for the last 44 h (n = 3−4). H) Heat map of individual ceramide species measured by direct infusion MS from 48 h TLR2-activated WT + BSA (vehicle), IL-10 KO + BSA (vehicle) or IL-10 KO + 25 uM 18:1 BMDMs (n = 3 per group). Scaled by row (lipid species). I) qPCR analysis of *Il6* and *Il1b* gene expression in 48 h TLR2-activated (50 ng/mL Pam3CysK4) WT BMDMs supplemented with CholPC (vehicle), Cer24:0 or Cer24:1 for the last 44 h of activation (n = 3 per group). All ceramides administered at a final concentration of 30uM. J) qPCR analysis of *Cxcl1* gene expression in 48 h TLR2-activated (50 ng/mL Pam3CysK4) WT BMDMs +/− DMSO (vehicle), 10 nM SCDi (Cay10566) +/− BSA or 25 uM 18:1 for the last 44 h. K) Total, saturated and unsaturated ceramide species measured by direct infusion MS from naïve or 48 h TLR2- activated WT BMDMs +/− DMSO (vehicle), 10 nM SCDi (Cay10566) +/− BSA or 25uM 18:1 for the last 44 h. L) Ceramide species measured by direct infusion MS from naïve or 48 h TLR2- activated WT BMDMs +/− DMSO (vehicle), 10 nM SCDi (Cay10566) +/− BSA or 25 uM 18:1 for the last 44 h. All experiments are reported as means ± SD from 3 independent experiments, unless noted otherwise. *P < 0.05; **P < 0.01, ***P < 0.005 (two-tailed unpaired Student’s *t* test). Source Data

To better understand how ceramide synthesis was regulated in *Il10*-KO cells, we examined all known genes required for sphingolipid metabolism from our matched lipidomics RNA-seq dataset (Extended Data Fig. 5a). Although we found no difference in levels of sphingolipid gene mRNA across genotype or activation status, we observed that expression of stearoyl-CoA desaturase 2 (*Scd2*) was significantly decreased in *Il10*-KO BMDMs (Fig. 3a and Extended Data Fig. 5a, bottom row). SCD2 is part of the SCD family (SCD1–4) of Δ9-desaturases that are responsible for the synthesis of MUFAs from saturated long chain fatty acids^29^. For example, SCDs convert saturated stearic acid (denoted 18:0) into mono-unsaturated oleic acid (denoted 18:1). MUFAs have been implicated in inflammation and sphingolipid homeostasis^30–33^, but the direct mechanisms of the regulation of these pathways by fatty acid desaturation remain unclear. Our previous studies found that SCD enzymatic activity regulates inflammation downstream of TLR activation^10^, leading us to hypothesize that altered MUFA synthesis could link inflammation and ceramide synthesis in IL-10-deficient macrophages. To determine whether the reduced *Scd2* gene expression observed in *Il10*-KO macrophages was sufficient to alter de novo MUFA synthesis, we performed stable-isotope tracer analysis on macrophages fed ^13^C-glucose for 48 h with or without TLR2 activation. In line with our previous work, we found that TLR2 stimulation increased de novo lipogenesis in wild-type BMDMs resulting in an increase in both synthesized and total 18:1 (Fig. 3b). Macrophages lacking IL-10 did not fully upregulate MUFA synthesis downstream of TLR2, resulting in reduced pools of synthesized and total 18:1 48 h after stimulation (Fig. 3b). By contrast, palmitate (16:0) synthesis was unchanged and total cellular amounts of palmitate and linoleic acid (18:2), an imported polyunsaturated fatty acid, were not decreased (Extended Data Fig. 5b,c). To determine whether altered MUFA content in *Il10*-KO BMDMs was important for inflammation, we activated wild-type, *Il10*-KO, and *Il10rb*-KO BMDMs with TLR2 ligand for 48 h and added back different bovine serum albumin (BSA)-conjugated free fatty acid species for the last 44 h. The addition of 18:1, which increased total 18:1 by approximately twofold (Extended Data Fig. 5d), reduced inflammatory gene expression in the *Il10*-KO or *Il10rb*-KO BMDMs (Fig. 3c and Extended Data Fig. 5e). Furthermore, addition of 24:1 (nervonic acid), an elongation product of 18:1, also reduced inflammatory gene expression in the *Il10*-KO BMDMs, whereas 16:0 and 18:2 did not (Fig. 3c and Extended Data Fig. 5e).

To test whether altered MUFA content in IL-10 deficient macrophages affected flux into the de novo sphingolipid synthesis pathway, we activated wild-type and *Il10rb*-KO BMDMs with TLR2 ligands in medium containing U-^13^C,^15^N-serine with BSA or BSA–18:1. Notably, addition of exogenous 18:1 could blunt enhanced U-^13^C,^15^N-serine incorporation into sphinganine in the *Il10rb*-KO macrophages (Extended Data Fig. 5f). Next, we performed shotgun lipidomics to examine how replenishment of 18:1 in *Il10*-KO BMDMs influenced ceramide homeostasis. We found that addition of 18:1 did not lower total ceramide abundance in the *Il10*-KO macrophages (Extended Data Fig. 5g), however, it significantly lowered the levels of saturated VLC ceramides, including Cer24:0 (Fig. 3d and Extended Data Fig. 5h). Notably, addition of 18:1 increased the amount of Cer24:1 and total mono-unsaturated ceramides (Fig. 3d and Extended Data Fig. 5h). Based on this result, we tested whether saturated and unsaturated VLC ceramides were equally responsible for heightened inflammation. In contrast to Cer24:0, exogenous Cer24:1 did not enhance inflammatory gene expression in macrophages (Extended Data Fig. 5i), indicating that only saturated VLC ceramides can drive an inflammatory phenotype.

Next, we sought to determine whether ceramide biosynthesis is influenced by macrophage de novo MUFA synthesis. In line with our *Il10*-KO data, we found that acute pharmacologic inhibition of SCDs (denoted SCDi) increased inflammatory gene expression, total ceramides and all ceramide species (Extended Data Fig. 5j–l). Similar to IL-10 deletion, the observed increase in inflammatory gene expression produced by SCDi could be mitigated by the addition of 18:1 (Extended Data Fig. 5j). Additionally, exogenous 18:1 blocked the excess accumulation of all saturated VLC ceramides in SCDi-treated macrophages (Extended Data Fig. 5k,l). Notably, addition of exogenous 18:1 did not alter VLC ceramide species with mono-unsaturated tails (with Cer24:1 being the most dominant unsaturated species) (Extended Data Fig. 5k,l). Thus, SCD activity and the pool size of 18:1 appears to specifically regulate the amounts of saturated VLC ceramides.

**Extended Data Fig. 6:**
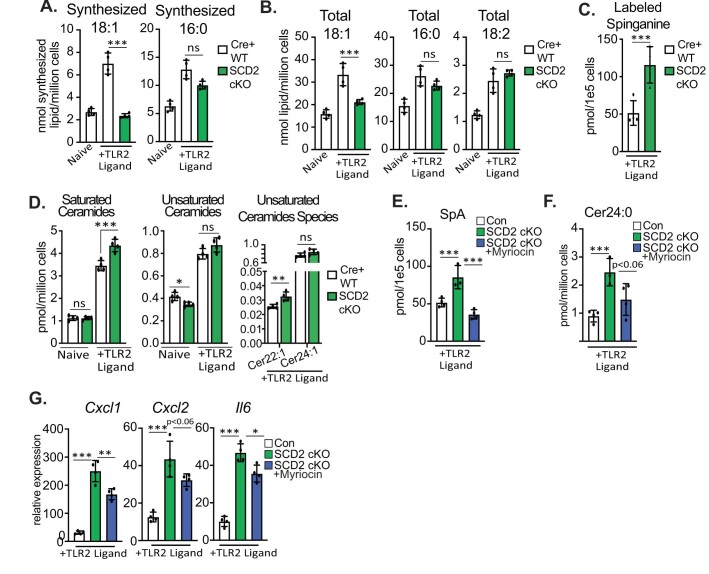
MUFAs regulates de novo ceramide synthesis. A. Net synthesis (nmol/million cells) of oleic acid (18:1) and palmitic acid (16:0) as measured by metabolic flux isotope tracer analysis in naïve or 48 h TLR2-activated (50 ng/mL Pam3CSK4) WT (white bars) or SCD2 cKO (green bars) BMDMs (n = 4 per group). B. Total cellular (nmol/million cells) abundance of oleic acid (18:1), palmitic acid (16:0) and linoleic acid (18:2) as measured by GCMS analysis of naïve or 48 h TLR2-activated (50 ng/mL Pam3CSK4) WT (white bars) or SCD2 cKO (green bars) BMDMs (n = 4 per group). C. Labeled sphinganine measured by LC-MS from 48 h TLR2-activated WT (white bars) or SCD2 cKO (green bars) BMDMs (n = 4 per group). D. Total Saturated and unsaturated ceramides species measured by direct infusion MS from naïve or TLR2-activated Cre+ WT (LysM Cre + /− SCD2^WT/WT^) and SCD2 cKO (LysM Cre + /− Scd2^flox/flox^) BMDMs (n = 4 per group). E. Total sphinganine measured by LC-MS from 48 h TLR2-activated Control (white bars), SCD2 cKO (green bars) or SCD2 cKO + 10 nM Myriocin (blue bars) BMDMs. Myriocin was spiked into culture for last 24 h of 48 h stim. (n = 3−4 per group). F. Cer24:0 in measured by LC-MS from 48 h TLR2-activated Control (white bars), SCD2 cKO (green bars) or SCD2 cKO +10 nM Myriocin (blue bars) BMDMs. Myriocin was spiked into culture for last 24 h of 48 h stim. (n = 3−4 per group). G. qPCR analysis of inflammatory gene expression from 48 h TLR2 activated Control (white bars), SCD2 cKO (green bars) or SCD2 cKO + 10 nM Myriocin (blue bars) BMDMs. Myriocin was spiked into culture for last 24 h of 48 h stim. (n = 4 per group). All experiments are reported as means ± SD from 3 independent experiments, unless noted otherwise. *P < 0.05; **P < 0.01, ***P < 0.005 (two-tailed unpaired Student’s *t* test). Source Data

Genetic deletion of *Scd2* is embryonic lethal in mice^29^. To determine the influence of SCD2 on inflammation in vivo, we generated *Scd2*^*flox/flox*^ mice and crossed them to LysM-*cre*^*+*^ mice to generate mice with myeloid-specific deletion of SCD2 (denoted *Scd2*-cKO). Analogous to *Il10*-KO BMDMs, *Scd2*-cKO BMDMs exhibited a similar reduction in synthesized and total 18:1, without affecting synthesized 16:0, total 16:0 and total 18:2 lipid species (Extended Data Fig. 6a,b). Notably, *Scd2*-cKO macrophages showed increased synthesis of sphinganine (Extended Data Fig. 6c) and accumulation of total and saturated VLC ceramides (Fig. 3e and Extended Data Fig. 6d), similar to *Il10*-KOs. Similarly, we observed increased inflammatory gene expression in *Scd2*-cKO macrophages in response to TLR2 activation that could be reduced by the addition of 18:1 or 24:1, but not 18:2 (Fig. 3f). To validate whether ceramides were important for enhanced inflammatory gene expression, we treated TLR2-activated *Scd2*-cKO BMDMs with myriocin, an inhibitor of serine palmitoyl transferase long chain base subunit 2 (SPTLC2). Myriocin treatment reduced sphinganine levels (Extended Data Fig. 6e), produced a trending decrease in Cer24:0 (Extended Data Fig. 6f), and reduced some inflammatory gene expression without affecting cell viability (Extended Data Fig. 6g and data not shown). These data suggest that flux into the ceramide de novo synthesis pathway is important for inflammatory phenotypes in SCD2-deficient macrophages. Together, these findings suggest that an inability to upregulate MUFA synthesis is a major underlying cause for accumulation of saturated ceramides and increased inflammation in IL-10 deficiency.

**Extended Data Fig. 7:**
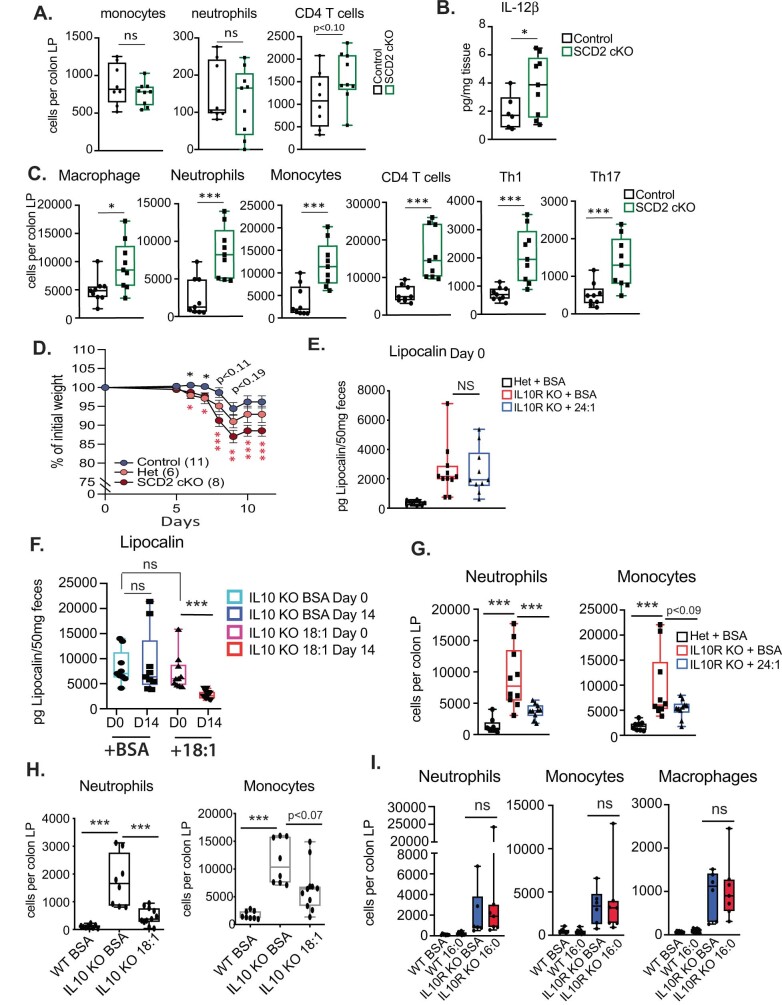
SCD2 and MUFAs regulate colonic inflammation. A) Flow cytometry based immune cell profiling of the colonic lamina propria from Control (LysM Cre +/−) and SCD2 cKO (LysM Cre +/− Scd2^flox/flox^) naïve male mice (n = 8−9 per genotype). B) ELISA analysis of IL-12B (IL12p40) from colonic explants of naïve Control (LysM Cre +/−) and SCD2 cKO (LysM Cre +/− Scd2^flox/flox^) male mice incubated ex vivo for 24 h. (n = 6−9 per genotype). C) Flow cytometry based immune cell profiling of the colonic lamina propria from Control (LysM Cre +/−) and SCD2 cKO (LysM Cre +/− Scd2^flox/flox^) female mice harvested on day 12 of DSS challenge (n = 9 per genotype). D) Weight loss measured during DSS-induced colitis in Control (LysM Cre +/−), SCD2 conditional heterozygous (Het) (LysM Cre +/−, Scd2 flox/WT) and SCD2 cKO (LysM Cre +/− Scd2 flox/flox) female mice (n = 6-11 per group). Red stars indicated statistical differences between Control and SCD2 cKO mice, while black stars represent statistical differences between Control and Het mice. There is no statistical difference in weight loss between SCD2 cHet and SCD2 cKO animals. Error bars represent Standard Error of the Mean (SEM). E) ELISA analysis of lipocalin from feces of Day 0: heterozygous (het) vs IL-10R KO mice, (all male mice). F) ELISA analysis of lipocalin from feces of Day 0: IL-10 KO female mice versus Day 14: IL-10 KO mice gavaged with BSA or BSA-18:1 (all female mice). G) Flow cytometry based myeloid cell profiling of the colonic lamina propria from heterozygous (het) mice gavaged with BSA, IL-10R KO mice gavaged with BSA or IL-10R KO mice gavaged with BSA-24:1 (all male mice). H) Flow cytometry based myeloid cell profiling of the colonic lamina propria from WT mice gavaged with BSA, IL-10 KO mice gavaged with BSA or IL-10 KO mice gavaged BSA-18:1 (all female mice). I) Flow cytometry based myeloid cell profiling of the colonic lamina propria from heterozygous (het) mice gavaged with BSA, IL-10R KO mice gavaged with BSA or IL-10R KO mice gavaged with BSA-16:0 (all male mice). All experiments are reported as means ± SD from 3 independent experiments, unless noted otherwise. *P < 0.05; **P < 0.01, ***P < 0.005 (two-tailed unpaired Student’s *t* test). Source Data

In line with our in vitro data, *Scd2*-cKO mice exhibited a basal elevation of faecal lipocalin compared with cohoused *cre*^*+*^ littermate controls (Fig. 3g). Additionally, there were increased numbers of macrophages in the colon of *Scd2*-cKO mice, with no difference in neutrophil or monocyte populations (Fig. 3h and Extended Data Fig. 7a; for gating strategy, see Supplementary Fig. 2). *Scd2*-cKO mice also had increased numbers of IFNγ^+^ and IL-17A^+^CD4^+^ T cells (Fig. 3h), indicating that myeloid-specific SCD2 expression affects T cell activation in the colon. In support of this idea, ex vivo colonic explants from *Scd2*-cKO mice produced more soluble IL-12p40 compared with tissue collected from cohoused *cre*^*+*^ littermate controls (Extended Data Fig. 7b). We hypothesized that the increased basal inflammation found in the *Scd2*-cKO mice might predispose these mice to models of chemically-induced colitis, such as dextran sodium sulfate (DSS). Similar to previously published studies in IL-10 deficient mice^4^, we found that DSS-treated *Scd2*-cKO mice lost significantly more weight than the cohoused littermate *cre*^*+*^ control mice, and had significantly increased numbers of infiltrating myeloid and lymphoid immune cells in the colon lamina propria (Fig. 3i and Extended Data Fig. 7c). SCD2 conditional heterozygous mice had an intermediary phenotype in response to DSS-induced colitis (Extended Data Fig. 7d). In sum, the phenotypes observed in SCD2-deficient macrophages and mice recapitulate phenotypes in macrophages and mice lacking IL-10 signalling.

Since exogenous MUFAs could limit inflammation in IL-10 deficient macrophages, and loss of myeloid-specific SCD2 resulted in increased colonic inflammation, we reasoned that exogenous MUFAs could reduce colonic inflammation in the *Il10*-KO or *Il10rb*-KO mice. To test this, we started with two groups of *Il10rb*-KO mice and two groups of *Il10*-KO mice that had similar levels of colonic inflammation, as measured by faecal lipocalin content (Extended Data Fig. 7e,f). For each genotype, one group of mice was orally gavaged with BSA, the carrier protein used to solubilize free fatty acids, or with BSA conjugated to 18:1 or 24:1. Mice were gavaged for 14 days before colonic tissue was collected for immune cell analysis and cytokine secretion. Markedly, MUFA gavage significantly reduced faecal lipocalin compared with BSA alone (Extended Data Fig. 7f). Furthermore, MUFA gavage reduced myeloid and lymphoid immune cell populations in the colon and reduced secretion of IL-6 and IL-12p40 from colonic explants (Fig. 3j,k and Extended Data Fig. 7g,h), at levels similar to those resulting from ablation of CerS2 (Fig. 2c and Extended Data Fig. 5a). Conversely, 16:0 gavage did not affect myeloid cell infiltration in wild-type or *Il10rb*-KO mice (Extended Data Fig. 7i). Together, these data support the idea that upregulation of MUFA synthesis in macrophages serves as a negative feedback mechanism downstream of IL-10 signalling to dampen inflammation, and that MUFA availability in macrophages has an important role in the control intestinal inflammation.

## VLC ceramides do not activate the inflammasome

**Extended Data Fig. 8:**
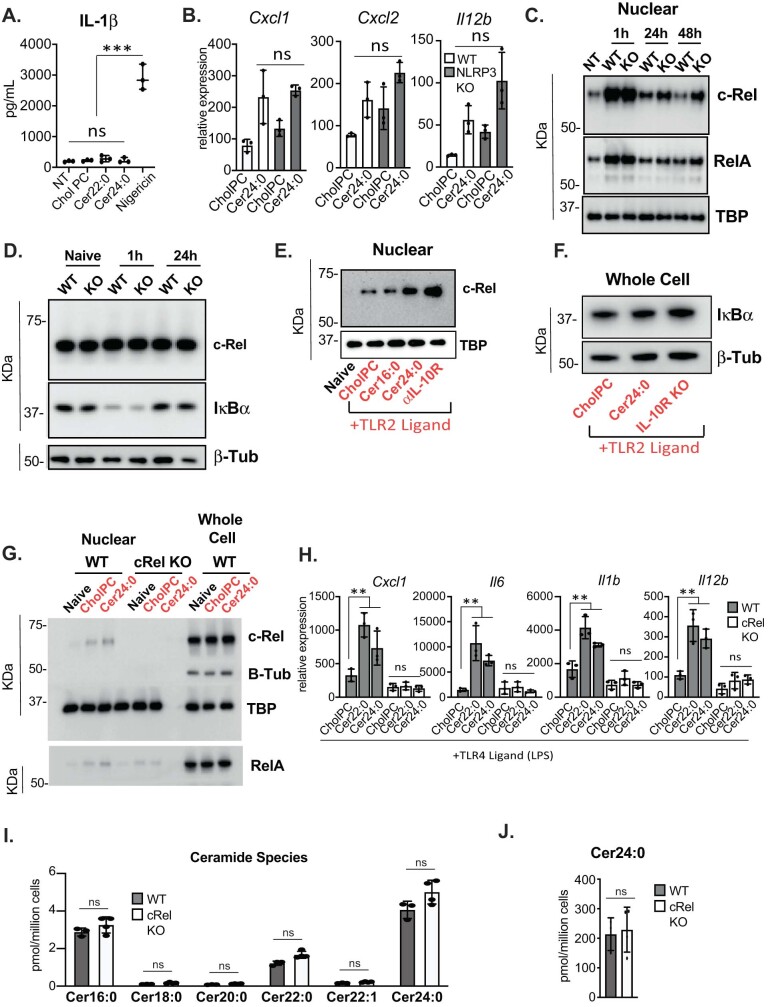
c-Rel mediated ceramide induced inflammation. A) ELISA analysis of IL-1B from the supernatants of WT BMDMs activated with TLR2 ligand, treated with CholPC (vehicle), Cer22:0, Cer24:0 for 48 h. Nigericin control was 1 h incubation after 48 h TLR2 ligand priming. All lipids were delivered at a final concentration of 30uM. B) qPCR analysis of inflammatory gene expression in WT or NLRP3 KO BMDMs activated with TLR2 ligand (50 ng/mL Pam3CysK4) for 48 h. TLR2-activated macrophage were incubated with Cholesteryl:Phosphatidylcholine (CholPC) lipid sheets alone or with CholPC loaded with ceramide 24:0 (Cer24:0) for the last 44 h of the activation (n = 3 for each group). All lipids administered at a final concentration of 30 uM. C) Western blot analysis of nuclear extracts from naïve, 1 h, 24 h, and 48 h TLR2-activated WT and IL-10R KO BMDMs for RelA, cRel and TBP (loading control). Representative of 2x individual experiments. D) Western blot analysis of whole cell lysates from naïve, 1 h or 24 h TLR2 activated WT and IL-10R KO BMDMs for c-Rel, Iκβα and β-tubulin (loading control). E) Western blot analysis of nuclear extracts from naïve or 24 h TLR2-activated WT peritoneal macrophage plus CholPC (vehicle), Cer16:0, Cer24:0 or anti-IL-10R neutralizing antibody (5 ug/mL) for the last 20 h of the activation for cRel and TBP (loading control). Representative of 4x individual experiments. F) Western blot analysis of whole cell lysates from 24 h TLR2 activated WT BMDMs treated with CholPC or 30 uM Cer24:0 or IL-10R KO BMDMs for Iκβα and β-tubulin (loading control). Representative of 2x individual experiments. G) Western blot analysis of nuclear extracts and whole cell lysates from naïve or 48 h TLR2-activated (indicated in Red) WT or c-Rel KO BMDMs plus CholPC (vehicle) or 30 uM Cer24:0 for the last 44 h of activation for RelA, c-Rel with B-Tubulin and TBP as loading controls. RelA was blotted in parallel with cRel and, B Tub and TBP. Representative of 2x individual experiments. H) qPCR analysis of inflammatory gene expression in 48 h TLR4-activated (50 ng/mL LPS) WT or cRel KO BMDMs plus CholPC (vehicle), 30 uM Cer22:0 or 30 uM Cer24:0 for the last 44 h of activation (n = 3 per group). I) Ceramide species in WT and c-Rel KO BMDMs activated with TLR2 ligand (50 ng/mL Pam3CysK4) for 48 h measured by direct infusion MS (n = 4). J) Total Cer24:0 after exogenous 30 uM Cer24:0 addback to WT or c-Rel KO BMDMs activated with TLR2 ligand (50 ng/mL Pam3CysK4 for 48 h measured by direct infusion MS (n = 3−4). All experiments are reported as means ± SD from 3 independent experiments, unless noted otherwise. *P < 0.05; **P < 0.01, ***P < 0.005 (two-tailed unpaired Student’s *t* test). Source Data

Next, we sought to determine the mechanism by which VLC ceramides enhance inflammation. Previous studies have suggested that the NLRP3 inflammasome may be important for ceramide-induced pathology in adipose tissue^34^. To test whether VLC ceramides induced inflammasome activation, we performed ELISAs to detect cleaved IL-1β in the supernatants of macrophages treated with VLC ceramides. Despite an increase in *Il1b* mRNA levels in response to VLC ceramides (Fig. 1g), neither Cer22:0 nor Cer24:0 induced IL-1β cleavage and secretion, in contrast to the strong induction of IL-1β cleavage and secretion induced by nigericin treatment^35^ (Extended Data Fig. 8a). Moreover, loss of NLRP3 activity did not influence VLC ceramide-mediated enhancement of inflammatory gene expression in BMDMs (Extended Data Fig. 8b), indicating that the NLRP3 inflammasome is not involved in ceramide-mediated inflammation in this system. These observations are consistent with previously published data indicating that de novo ceramide biogenesis is not required for NLRP3 inflammasome activation^36^.

## REL is required for ceramide-induced inflammation

**Fig. 4:**
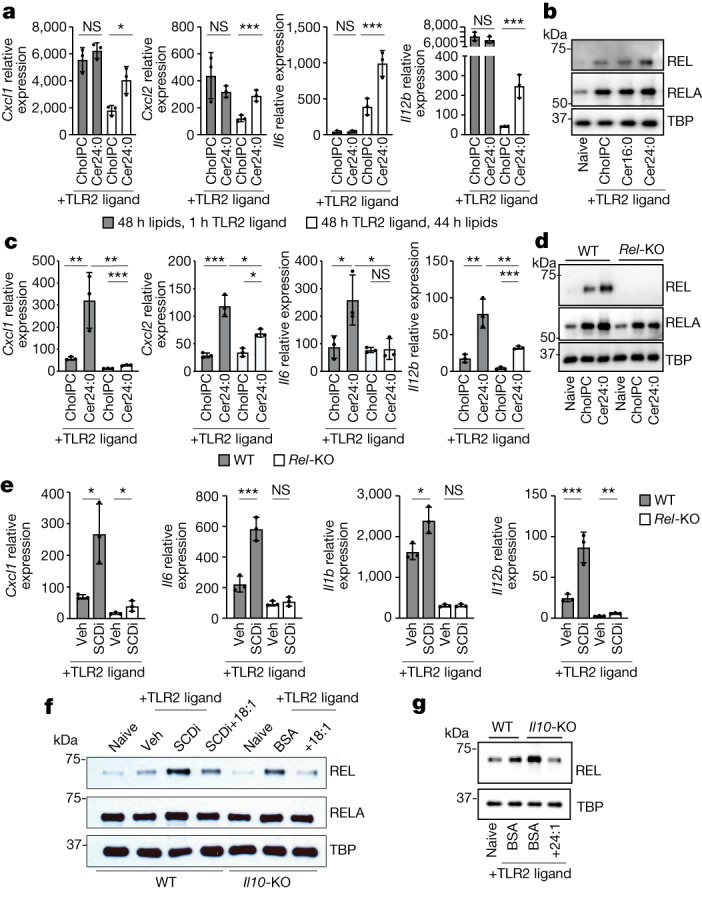
REL is required for ceramide-mediated induction of inflammatory gene expression. **a**, qPCR analysis of inflammatory gene expression in BMDMs pretreated with 30 μM Cer24:0 for 48 h, followed by 1 h activation with TLR2 ligand (50 ng ml^−1^ Pam3CSK4) versus 48 h TLR2 activation with 44 h 30 μM Cer24:0 treatment (*n* = 3). **b**, Western blot analysis of RELA, REL and TBP (loading control) from nuclear extracts from naive or 48 h TLR2-activated wild-type peritoneal macrophages plus cholPC (vehicle), Cer16:0, or Cer24:0 for the last 44 h of the activation. Representative of three individual experiments. **c**, qPCR analysis of inflammatory gene expression in 48 h TLR2-activated wild-type or *Rel*-KO BMDMs plus cholPC (vehicle) or 30 μM Cer24:0 for the last 44 h of activation (*n* = 3). **d**, Western blot analysis of RELA, REL and TBP (loading control) from nuclear extracts from naive or 48 h TLR2-activated wild-type or *Rel*-KO BMDMs plus cholPC (vehicle) or 30 μM Cer24:0 for the last 44 h of activation. RELA was blotted in parallel with REL and TBP. Representative of two individual experiments. **e**, qPCR analysis of inflammatory gene expression in 48 h TLR2-activated wild-type or *Rel*-KO BMDMs with or without 10 nM SCDi (Cay10566) for the last 44 h of activation (*n* = 3). Veh, vehicle. **f**, Western blot analysis of REL, RELA and TBP (loading control) in nuclear extracts from naive or 48 h TLR2-activated wild-type or *Il10*-KO BMDMs plus SCDi, BSA or 25 μM BSA–18:1 for the last 44 h of the activation. Representative of three individual experiments. **g**, Western blot analysis of nuclear extracts from naive or 48 h TLR2-activated wild-type or *Il10rb*-KO BMDMs plus BSA or 25 μM BSA–24:1 for the last 44 h of the activation for RELA, REL and TBP (loading control). Representative of two experiments. All experiments are reported as mean of biological replicates ± s.d. *P < 0.05, **P < 0.01, ***P < 0.005 (two-tailed unpaired Student’s *t*-test). Source Data

It is widely accepted that IL-10 signalling acts at the level of transcription to repress inflammatory gene expression; however, the exact steps that govern this process remain unclear^37^. Consistent with this idea, we observed elevated nuclear levels of the NF-κβ family transcription factors REL (also known as cRel) and RELA (also known as p65) only at late timepoints (24 and 48 h) after TLR2 activation in *Il10*-KO macrophages, without alterations in initial transcription factor nuclear translocation (Extended Data Fig. 8c) or IκBα degradation and re-accumulation patterns^38,39^ (Extended Data Fig. 8d). This led us to hypothesize that increased VLC ceramides may have a role only in late-stage inflammatory responses that are characteristic of IL-10 deficiency. To test this idea, we pretreated BMDMs with cholPC or Cer24:0 for 48 h, followed by a brief 1 h TLR2 stimulation. Inflammatory gene expression patterns of cells pretreated with lipid were compared to BMDMs activated with TLR2 ligands for 48 h and with exogenous Cer24:0 for the last 44 h of activation (as in our previous experiments). Pretreatment with Cer24:0 did not alter early induction of inflammatory genes, but resulted in prolonged expression at later timepoints (Fig. 4a). We also found that Cer24:0 treatment, but not Cer16:0 treatment, resulted in increased nuclear REL and RELA after 24 h and 48 h TLR2 activation (Fig. 4b and Extended Data Fig. 8e), without affecting total cellular amounts of IκBα (Extended Data Fig. 8f), REL or RELA (Extended Data Fig. 8f,g), similar to results from IL-10 deficiency (Extended Data Fig. 8c–e).

RELA and REL are related transcription factors that can homodimerize with themselves or heterodimerize with one another to induce inflammatory gene expression^40–42^. RELA is absolutely required for the initial induction of inflammatory cytokines and chemokines in activated macrophages and other immune and non-immune cells^42–45^. REL is thought to have a more limited role in inflammation, in which only TLR-mediated induction of *Il12a* and *Il12b* is strongly dependent on REL in myeloid cells^46^. We observed dysregulation of *Il12b* and *Il12a* in most of our in vitro and in vivo experiments, leading us to hypothesize that saturated VLC ceramides (such as Cer24:0) influence inflammation by specifically modulating REL activity. In line with this idea, *Rel*-KO BMDMs displayed reduced inflammatory gene expression in response to VLC ceramides when activated with TLR2 or TLR4 ligands (Fig. 4c and Extended Data Fig. 8h). Markedly, endogenous ceramide levels and exogenous ceramide uptake were not altered by REL deficiency, suggesting that REL is downstream of ceramide-induced inflammation (Extended Data Fig. 8i,j). Markedly, we found that loss of REL abrogated Cer24:0-mediated induction of RELA (Fig. 4d and Extended Data Fig. 8g), suggesting that the pool size of VLC ceramides may directly regulate REL, and that enhanced RELA nuclear localization is either dependent on dimerization with REL or is mediated by signals downstream of REL activation.

**Extended Data Fig. 9:**
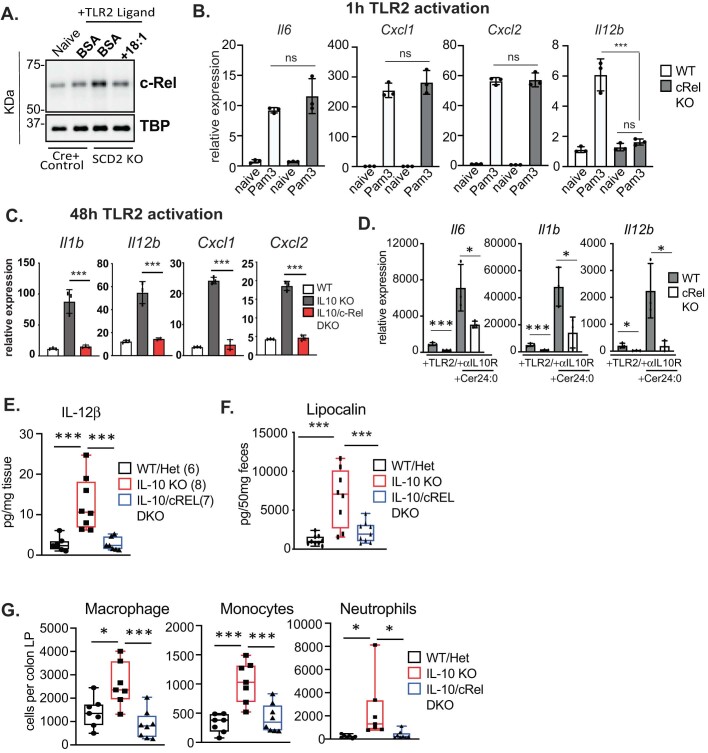
c-Rel is required for late stage inflammation in vitro and in vivo. A. Western blot analysis of nuclear extracts from naïve or 48 h TLR2-activated Cre+ WT (LysM Cre +/− SCD2^WT/WT^) and SCD2 cKO (LysM Cre +/− Scd2^flox/flox^) plus BSA or BSA-18:1 for the last 44 h of the activation for cRel and TBP (loading control). Representative of 2x individual experiments. B. qPCR analysis of inflammatory gene expression in 1 h TLR2-activated (50 ng/mL Pam3CysK4) WT or c-Rel KO BMDMs (n = 3 per group). C. qPCR analysis of inflammatory gene expression in 48 h TLR2-activated (50 ng/mL Pam3CysK4) WT, IL-10 KO or IL-10/c-Rel DKO BMDMs (n = 3 per group). D. qPCR analysis of inflammatory gene expression in 48 h TLR2-activated WT or cRel KO BMDMs + anti-IL-10R neutralizing antibody (5 ug/mL), +/− Cer24:0 for the last 44 h of the activation (n = 3 per group). E. ELISA analysis of IL-12B (IL12p40) from colonic explants of WT, IL-10 KO or IL-10/cREL DKO male mice incubated ex vivo for 24 h. (n = 7−8). F. ELISA analysis of fecal lipocalin from WT, IL-10 KO and IL-10/c-Rel DKO mice (n = 7−8). G. Flow cytometry based Immune cell profiling of the colonic lamina propria from naive WT, IL-10 KO or IL-10/c-Rel DKO male mice (n = 7−8). All experiments are reported as means ± SD from 3 independent experiments, unless noted otherwise. *P < 0.05; **P < 0.01, ***P < 0.005 (two-tailed unpaired Student’s *t* test). Source Data

In complementary studies, we found that SCDi-mediated induction of inflammatory genes is dependent on REL (Fig. 4e), and that SCDi treatment or conditional knockout of *Scd2* can induce REL nuclear localization in macrophages, which can be mitigated by addition of exogenous 18:1 (Fig. 4f and Extended Data Fig. 9a). Moreover, nuclear localization of REL in *Il10-*KO or *Il10rb*-KO macrophages was also limited by addition of 18:1 (Fig. 4f) or 24:1 (Fig. 4g), whereas changes in localization of RELA were less affected. Together, these data indicate that REL has an essential role in VLC ceramide-mediated induction of inflammatory genes, and is largely responsible for enhanced inflammatory phenotypes found in IL-10 or MUFA-deficient mice and macrophages.

Owing to its role in lipid-mediated inflammation, we hypothesized that REL may have a broader role in regulating inflammation than previously described. Consistent with previous results^46,47^, we found that REL is required for induction of *Il12b* gene expression, but not other cytokine or chemokine genes, after 1 h TLR2 activation (Extended Data Fig. 9b). However, after 48 h, genetic deletion of *Rel* in *Il10*-KO mice (*Il10*/*Rel-*DKO mice) completely ablated the enhanced inflammatory gene expression back to wild-type levels (Extended Data Fig. 9c), indicating that REL is critical for late-stage inflammatory gene expression in *Il10*-KO macrophages. Furthermore, REL was required for full induction of inflammatory gene expression in response to IL-10R neutralization alone and in combination with Cer24:0 addition (Extended Data Fig. 9d) after 48 h of TLR2 activation, which supports the hypothesis that REL is crucial for saturated VLC ceramide-driven inflammation caused by IL-10 deficiency. Furthermore, *Il10*/*Rel-*DKO mice exhibit reduced colonic secretion of IL-12p40 (Extended Data Fig. 9e), reduced faecal lipocalin (Extended Data Fig. 9f) and reduced myeloid cell numbers in the colon lamina propria compared with *Il10*-KO mice (Extended Data Fig. 9g), indicating that REL is critical for maintaining unresolved inflammation in IL-10-deficient macrophages and mice.

## Discussion

In this study we mechanistically delineate crosstalk between known transcriptional regulators of the immune response, lipid metabolism and intestinal homeostasis. Here, we uncover a previously undescribed mechanism by which the anti-inflammatory cytokine IL-10 regulates the duration of inflammation. Specifically, in the absence of IL-10 signalling, we find enhanced flux into the de novo sphingolipid synthesis pathway, resulting in the accumulation of VLC ceramides that promote inflammatory gene expression. Unexpectedly, we found that altered sphingolipid production was not mediated by transcript levels of genes encoding enzymes in the synthesis pathway (Extended Data Fig. 5a). Instead, changes in ceramide biogenesis were traced to the ability of IL-10 to control *Scd2* gene expression and subsequent synthesis of long chain MUFAs (Fig. 3a,b). Notably, pharmacologic inhibition or genetic deletion of SCD2 engaged the de novo ceramide synthesis pathway in a similar manner to IL-10 deletion (Fig. 3e and Extended Data Figs. 5k,l and 6c–f). There are few known checkpoints that regulate flux into the de novo ceramide synthesis pathway, and how de novo MUFA synthesis antagonizes this pathway remains unclear. One possibility is that when MUFA synthesis is limited, this results in an increase in saturated fatty acid precursors such as palmitate. The condensation of palmitoyl-CoA and serine is the first step in ceramide de novo synthesis pathway and is required for the generation of all downstream sphingolipid metabolites. Indeed, IL-10 deficiency slightly increases total cellular palmitate in macrophages (Extended Data Fig. 5c). However, genetic deletion of SCD2, which also blunts MUFA synthesis and increases total ceramides, does not affect total or synthesized palmitate (Extended Data Fig. 6a,b), suggesting that other mechanisms of regulation are more likely. Notably, we find that SCDi treatment or conditional knockout of *Scd2* increases the amount of most ceramide species regardless of saturation of the N-acylated tail (Extended Data Figs. 5l and 6d). This suggests that MUFAs are not limiting with regard to the ceramide acyl tail and may have a broader role in regulating flux into ceramide biogenesis outside of substrate availability. Thus, another possibility is that IL-10-mediated MUFA synthesis may engage the activity of orosomucoid (ORM) family proteins. ORM proteins antagonize serine palmitoyl transferases such as SPTLC2 to reduce flux into the sphingolipid pathway^48^. Notably, ORMs are known to influence airway inflammation and have been reported to be controlled by other metabolic programmes^49,50^. Further biochemical analysis will be required to fully examine what factors are required for the effect of IL-10 signalling and MUFAs on ceramide de novo synthesis.

Many previous studies examining the influence of ceramides on inflammation and apoptosis utilize short chain C2 ceramide analogues that are not endogenously synthesized by mammalian cells. Here we focus on long chain and VLC ceramides that are produced endogenously by immune cells, and find that exogenous addition of long chain and VLC ceramides—in contrast to C2 ceramide—do not appear to induce cell death or drive inflammasome activation. Additionally, we observe that only saturated VLC ceramides, but not their long chain counterparts, can enhance macrophage inflammation (Fig. 1g and Extended Data Fig. 2g,h), providing experimental evidence for results predicted in silico^51^. Furthermore, we identify that the ‘desaturation status’ of the tails of VLC ceramides is an additional feature that is critical for modulating the magnitude of macrophage inflammation (Extended Data Fig. 5i). These findings suggest a remarkable degree of specificity in how VLC ceramides convey information to the inflammation machinery, and it will be important for in future studies to focus on how saturated but not unsaturated VLC ceramides mediate inflammatory gene expression. Additionally, further studies are required to explore how downstream sphingolipid metabolites affect inflammation. For example, VLC ceramide treatments increase VLC hexosyl ceramides (Extended Data Fig. 2c), which are thought to be ligands for CD1d, a noncanonical MHCII required for activation of invariant natural killer T cells^52–54^. Further, it remains unknown how IL-10 signalling affects the generation of sphingosine-1-phosphate, a highly bioactive lipid that is known for this critical role in adaptive immune cell trafficking^55^ (reviewed in refs. ^56,57^). Thus, further studies are required to fully elucidate the role IL-10 signalling and the sphingolipidome in macrophages. It is also worth noting that loss of IL-10 signalling reduces most saturated and all mono-unsaturated sphingomyelins (Fig. 1d and Extended Data Fig. 1f). This could be due to a reduction in MUFAs (Fig. 3b), perturbed endoplasmic reticulum-to-Golgi transport that is required for sphingomyelin synthesis, or enhanced sphingomyelin degradation. Enhanced sphingomyelin degradation has been observed in response to inflammatory cytokine signalling in immune cell types^17,18^, but the exact mechanism for how increased sphingomyelin degradation influences inflammation remains poorly understood. Further studies are required to determine if and how IL-10 signalling affects sphingolipid salvage pathways.

We found that the persistent inflammation observed in response to VLC ceramides or the absence of MUFA synthesis could be traced to sustained activity of the NF-κB family member REL. Crosstalk between IL-10 and REL has previously been studied in macrophages^58,59^, but the observation that REL transcriptional activity can be influenced by fatty acid desaturation downstream of IL-10 signalling strengthens the idea that manipulation of fatty acid homeostasis is directly linked with immune cell activation. We also provide strong evidence that REL is pivotal for persistent inflammatory gene expression in the absence of IL-10 signalling and is critical for inflammatory gene expression outside of *Il12a* and *Il12b*, its prototypic targets in myeloid cells^46,47^. Thus, identification of lipid sensor signalling pathways that regulate REL activity may serve as new targets to modulate the aberrant inflammation observed in a broad array of metabolic and sterile inflammatory diseases. The exact mechanism by which saturated VLC ceramides specifically direct REL cellular location remains unclear and will be an important topic of future studies. Why REL nuclear location is more greatly affected than RELA in response to VLC ceramides also remains unclear—this may be due to inflammatory kinetics, transcription factor-specific post-translational modifications, or unknown signalling cascades that target specific NF-κB family members. Further studies will be required to fully understand how VLC ceramides engage with REL activation.

Finally, we find that genetic reduction of VLC ceramides or oral gavage with MUFAs can reduce colonic inflammation found in IL-10- or IL-10R-deficient mice (Fig. 2c–f and Extended Data Figs. 3i–k, 4a–d and 7g,h), suggesting that regulation of de novo lipid synthesis pathways is important for the maintenance of colonic homeostasis and health. Humans with deleterious mutations in IL-10 or IL-10R present with severe, life-threatening enterocolitis within the first few months of life. These individuals do not respond positively to standard forms of immune suppression such as anti-TNF biologicals or steroids. Besides haematopoietic stem cell transplant, there is no effective treatment option for IBD in people who lack functional IL-10 signalling^2^. Intriguingly, dietary intervention with the Mediterranean diet, which is high in unsaturated fats, has been shown to reduce disease parameters in patients with IBD in as little as six months^60^. A key component of the Mediterranean diet is olive oil, which is high in MUFAs. Thus, it is tempting to speculate that the benefits of the Mediterranean diet may be dependent, in part, on MUFA-mediated repression of colonic inflammation via changes in VLC ceramide metabolism. Indeed, our mouse data strongly suggest that oral delivery of exogenous MUFAs can beneficially curb inflammation in the colon and may serve to establish new treatments for colitis focused on fatty acid ‘metabolite correction’.

## Methods

### Mouse Strains

*Il10*-KO (JAX 002251) and *Il10rb*-KO (JAX 005027) mice were purchased from Jackson Laboratories. Both knockout strains were crossed to wild-type C57BL/6 (JAX 000664) to generate heterozygous mice. All future cohorts of mice were generated from heterozygote × heterozygote breeding to minimize microbial diversity. Cohorts of mice were aged in cohoused cages with three wild-type mice and three knockout mice unless noted otherwise. LysM-*cre*^*+/−*^ (JAX 004781) mice were also purchased from Jackson Laboratory. S*cd2*^*flox/flox*^ mice and *Cers2-*KO mice were generated in our in-house CRISPR Core and are available upon request. For bone marrow chimeras, donor bone marrow (250,000–500,000 cells per mouse) was transferred into CD45.1 PepBoy (Jax 002014) recipient mice. Mice were engrafted for 10 weeks prior to experimental use. All animal experimentation was performed in compliance with Yale Institutional Animal Care and Use Committee protocols. Mice were housed with 14 h light:10 h dark cycles in rooms maintained at 21.5 °C with 62% room humidity.

For in vivo mouse experiments, sample sizes were determined based upon the availability of mice and were the largest possible. Mice were not randomized since mice were analysed based upon their genotype. Investigators were not blinded to group allocation during data collection or analysis. Blinding were not relevant to our study given that most observations made in this study were flow cytometry based and the genotypes of the mice were confirmed before and after analysis.

### DSS colitis

Mice were given 1.5% DSS in their drinking water for 5 days, before changing back to normal water bottles. Mice were weighted through the duration of the experiment and euthanized at day 12. Mice were 10 weeks or older with a starting weigh of 20 g or higher.

### Ex vivo mouse cells

For BMDMs, bone marrow was differentiated into macrophages in DMEM containing 10% FBS (Sigma), 5% M-CSF conditioned medium or 50 ng ml^−1^ M-CSF (Biolegend) (results did not differ), 1% penicillin-streptomycin (Gibco), 1% glutamine (Invitrogen) 0.5% sodium pyruvate (Invitrogen) for 7–9 days prior to experimental use. Peritoneal macrophages were collected after 96 h thioglycallate treatment. Macrophages were washed, counted, plated in the medium described above, activated by TLR agonists and maintained in culture for 24–48 h.

### Reagents

Cells were treated with 50 ng ml^−1^ Pam3CSK4 (Invivogen tlrl-pms). CholPC (700123), Cer16:0 (860516**)**, Cer22:0 (860525), Cer24:0 (860524) or Cer24:1 (860525) (Avanti Lipids). All ceramides were used at a final concentration of 30 μM. Myriocin (Cayman Chemicals) was solubilized in DMSO. Working solution was generated by 1:1,000 dilution for a final working solution of 10 nM for the last 24 h of TLR activations.

#### Preparation of ceramide:cholPC

This method was adapted from ref. ^61^. Ceramides and cholPC were dissolved into 100% chloroform to 12.5 mM. Equal mixtures of ceramide and cholPC were mixed and dried under nitrogen. Lipids were rehydrated in PBS to 3.125 mM Cer:3.125 mM cholPC (or cholPC:blank), and sonicated at 55 °C for ~1 h until all precipitates were in solution. Lipid mixtures were added to cell cultures at 1:100 dilution for 31.25 μM final concentration for each ceramide species.

#### Preparation of 24% BSA

Six grams of fatty acid-free, endotoxin-low BSA (Sigma A8806**)** was gradually added to 17.5 ml 150 mM NaCl in a beaker at 37 °C while stirring. Once BSA was completely dissolved, pH was adjusted to 7.4 with 1 N NaOH (adding 1 M NaOH slowly in 20 μl increments, while stirring). This was repeated with incremental 10× NaOH dilutions slowly until the desired pH was achieved. Final volume was adjusted to 25 ml with 150 mM NaCl.

#### Preparation of BSA-conjugated free fatty acids

FFAs (Nucheck Prep) were dissolved 150 mM NaCl pH 7.4 to 25 mM final concentration (that is, 50 mg 18:1 was dissolved into 7 ml 150 mM NaCl plus 48 μl NaOH). Mixture was shaken vigorously, and heated at 65 °C until in solution. BSA solution (24%, ice cold) was finally combined with 25 mM oleic acid solution (room temp) in a 54:46 ratio to yield approximately 12.5 mM final concentration with pH 7.4. This was stored at −20 °C and mixed for 10 min at room temperature on a shaker before use.

#### Addition of FFAs into cell culture

FFAs were diluted into cell culture medium at 1:500 dilution for 25 μM final concentration. Free fatty acids were added 4 h post TLR stimulation.

#### Oral gavage of BSA–18:1

Mice (15–18 g, 5–6 weeks old) were gavaged for 14 days with 100 μl BSA alone or 6.25 μM BSA–18:1 (preparation described above, diluted 1:1 with PBS).

#### Inhibition of MUFA synthesis by SCDi

SCDi (Cay10566; Fisher NC0493687) was dissolved into DMSO to a final concentration of 10 μM. This solution was used immediately, only once, and did not undergo any freeze–thaw cycles (any freeze–thaw cycles killed the activity of this compound as measured by isotope tracer analysis). Working solution was generated by 1:1,000 dilution for a final working solution of 10 nM.

Cell viability was assessed with AOPI using the Nexcelom K2 cell counting system.

### ELISAs

Lipocalin (R&D), IL-6 (R&D) and IL-12b (Biolegend) ELISAs were preformed to manufacturer’s instructions.

### Ex vivo peritoneal macrophage collection

Wild-type and *Il10*-KO mice were treated with thioglycallate via intraperitoneal injection for 48 h, followed by intraperitoneal injection of 50 μg per mouse TLR2 ligand (Pam3CSK4) for an additional 48 h. Macrophages were collected by peritoneal lavage, counted, and prepared for mass spectrometry lipidomic analysis described below. A sample of ex vivo cells were assessed for purity by flow cytometry (97% myeloid for both wild-type and *Il10*-KO cells).

### Lipidomics analysis

Macrophages were cultured in 6-well dishes (Fisher 08-772-1B) and stimulated with TLR ligands as described above. Forty-eight hours after stimulation, cells were imaged for cell count as previously described^9^, scraped and spun down in PBS, and snap-frozen as cell pellets. A modified Bligh and Dyer extraction^62^ was carried out on samples. Prior to biphasic extraction, a 13 lipid subclass Lipidyzer Internal Standard Mix was added to each sample (AB Sciex, 5040156). In later experiments, internal standard mixture consisting of 70 lipid standards across 17 subclasses was added to each sample (AB Sciex 5040156, Avanti 330827, Avanti 330830, Avanti 330828 and Avanti 791642). In most recent experiments, a standard mixture of 75 lipid standards across 17 subclasses (Avanti 330820, Avanti 861809, Avanti 330729, Avanti 330727 and Avanti 791642) was added to each sample. Following 2 successive extractions, pooled organic layers were dried down in the Thermo SpeedVac SPD300DDA using ramp setting 4 at 35 °C for 45 min with a total run time of 90 min. Lipid samples were resuspended in 1:1 methanol:dichloromethane with 10 mM ammonium acetate and transferred to Robovials (Thermo 10800107) for analysis. Samples were analysed on the Sciex Lipidyzer Platform (Sciex 5500 with DMS and Shimadzu LC-30) for targeted quantitative measurement of 1100 lipid species across 13 subclasses. In later experiments, an expanded assay consisting of 1,400 targeted lipid species across 17 subclasses was used. Differential Mobility Device on Lipidyzer was tuned with SelexION tuning kit (Sciex 5040141) or EquiSPLASH LIPIDOMIX (Avanti 330731). Instrument settings, tuning settings, and multiuple reaction monitoring lists have been previously published^63^. Data analysis was performed on Lipidyzer software (v1.0). Later experiments were analysed with the Shotgun Lipidomics Assistant (SLA v1.3)^63^. Quantitative values were normalized to cell counts. PCA and heat maps were generated using guidelines described^64^.

### Detection of sphinganine

The detection and quantification of sphinganine were performed using an Agilent 6490 ESI-QQQ-MS/MS or an Agilent iFunnel 6550 quadrupole time of-flight (QTOF) mass spectrometer, fitted with an electrospray ionization (ESI) source (positive) coupled to an Agilent 1290 Infinity HPLC system. The target lipid was analysed utilizing a gradient program on a Phenomenex Kinetex 1.7 mm C18 100 Å (100 × 2.1 mm) (10% → 100% MeCN in water with 0.1% formic acid for 7 min, 0.3 ml min^−1^, column temperature: 50 °C). The detection with ESI-QQQ-MS/MS was conducted with the optimized collision energy at 20 V and facilitated by dynamic multiple reaction monitoring (MRM) mode with the mass transition *m/z* 302 → 284. The QTOF mass spectrometry detection was performed with the following source parameters: gas temperature 280 °C, drying gas 11 l min^−1^, nebulizer 40 psi, sheath gas temperature 350 °C, and sheath gas flow 11 l min^−1^.

For labelled sphinganine detection, cells were grown in serine-free medium (Teknova; serine-, glucose- and glycine-free) supplemented with 84 mg l^−1^ U^13^C^15^N-serine (Cambridge Isotope Laboratories 202407-34-9) for 48 h before lipid extraction and mass spec analysis as described above. Total and labelled (+3 Da) sphinganine was detected with Mass Hunter Software (Agilent) and normalized to a standard curve. Cold glucose and glycine were supplemented to normal DMEM levels of 4.5 g l^−1^ and 30 mg l^−1^, respectively.

### Isotope enrichment experiments

Day 8 differentiated BMDMs were transferred to complete medium containing 50% U^13^C-glucose with or without TLR stimulation for 48 h before collection. See ref. ^9^ for further details. Analysis of labelled fatty acids and cholesterol was performed as described^65^. The relative contributions of synthesis to the total cholesterol pool over the 48 h labelling period were determined by fitting the isotopologue distributions for cholesterol in a model similar to isotopomer spectral analysis as described^65^.

### Gene expression analysis

RNA was extracted from all cells with Trizol using manufacturer’s protocols. cDNA was synthesized with Applied Biosystems High Capacity cDNA Synthesis Kit as per manufacturer’s instructions (700 ng μl^−1^ RNA per cDNA synthesis reaction). qPCR was conducted on the BioRad qPCR machine using SYBR Green Master Mix (BioRad) and 0.5 μmol l^−1^ primers. Relative expression values are normalized to control gene (rRNA 36B4) and expressed in terms of linear relative mRNA values. Primer sequences are available upon request.

### Western blot antibodies

Murine REL (Santa Cruz SC-71) (dilution 1:1,000); RELA (Cell Signaling 8242) (dilution 1:1,000); TBP (Cell Signaling) (dilution 1:10,000); β-tubulin (Cell Signaling) (dilution 1:500); Iκβα (Cell Signaling) (dilution 1:1,000).

### Nuclear extract preparation

Two million BMDMs or peritoneal macrophages were incubated with ALLN at 37 °C for 15 min before collection. Cells were washed with PBS + ALLN, then transferred to Eppendorf tubes. Cells were incubated in 450 μl hypotonic buffer A (10 mM Hepes pH 7.9, 10 mM KCl, 0.1 mM EGTA, 0.1 mM EDTA + protease inhibitors) for 15 min on ice, then 20 μl of 10% NP-40 was added and cells for vortexed for 10 s to lyse the plasma membrane. Nuclei were collected by centrifugation (12,000*g*) for 5 min, and resuspended in 100 μl hypertonic buffer (20 mM Hepes pH 7.9, 420 mM NaCl, 1.5 mM MgCl2, 0.2 mM EDTA, 25% glycerol+ protease inhibitors). After thorough mixing, nuclei were incubated at 4 deg C on a rotator for 1 h. Samples were quickly vortexed, spun down at 12,000*g* for 5 min to separate the nuclear pellet. Nuclear extracts were collected and immediately frozen on dry ice or mixed with loading buffer, boiled for 10 min and run on an SDS–PAGE for immunoblot analysis.

### Immunoblots

Samples were normalized by cell number (Nexcelom K2 cell counting system) and lysed directly into 2× Laemmli loading buffer. Protein extracts were separated on gradient 4% to 12% Bis–Tris SDS–PAGE gel (Invitrogen) and then transferred to a nitrocellulose membrane (Amersham). After blocking for 1 h in a TBS containing 0.1% Tween 20 (TBST) and 5% nonfat milk, the membrane was probed with indicated antibodies diluted into TBST with 5% milk overnight. Membranes were washed 4× with TBST, followed by a 30-min room temperature incubation with secondary antibodies conjugated to horseradish peroxidase diluted in TBST plus 5% milk. Membranes were washed as before and then developed using Pierce ECL2 detection kit and imaged with Typhoon.

### Faecal lipocalin preparation

Faeces of each mouse were collected, weighed and dissolved in PBS plus 0.1% Tween. Faecal pellets were disrupted using a tube shaker for 5 min at full speed. Samples were spun down and the resulting supernatants were diluted 1:10 and utilized for lipocalin ELISAs.

### Isolation of immune cells from the colon lamina propria

Colons were collected, flushed with 10 ml PBS, and split lengthwise, and rinsed again in PBS. The epithelial fraction was removed with two 10 ml washes of epi wash buffer (1× HBSS, 5 mM EDTA, 1 mM DTT) at 37 °C for 20 min while shaking at 220 rpm. Colons were removed from epi wash buffer, washed in PBS and finely minced with a razor blade. Minced tissue was transfer into 5 ml digestion buffer (1× DMEM, 5% FBS, 1 mg ml^−1^ collagenase D, 0.5 mg ml^−1^ DNAase) and incubated at 37 °C for 60 min while shaking at 220 rpm. After digestion, samples were strained though at 70-μm filter and washed twice with 20 ml 1× DMEM + 5% FBS. Cells were then divided into five groups for different staining panels. Cells were stained with antibodies (1:400 dilution) and fixable viability dye (1:1,000 dilution) at 4 °C for 30 min in FACS buffer (2% FBS in PBS), washed twice, and run immediately on an LSR2 or fixed (BD Cytofix/CytoPerm 554722) for intracellular staining with cytokine antibodies (1:100 dilution). AccuCheck cell counting beads (Invitrogen PCB100) were utilized for cell number quantification. All flow cytometry data was collected on BD LSR2s with FACSDiva 7 software. Flow cytometry data were analysed with FlowJo (v9 to v10.6.1).

### Flow cytometry antibodies

From Biolegend: PerCPCy5.5-Cd45.2 (Clone 104), 109828; AF700-Cd45.2 (Clone 104), 109822; BV421 CD45.1 (clone A20), 110731; BV711 Cd11b (Clone M1/70), 101242 APC CD11c (Clone N418), 117310; AF700 MHCII (I-A/I-E, clone M5/114.15.2), 107628; PE CD64 (clone X54-5/7.1), 139303; APC Cy7 Epcam (clone G8.8), 118218; AF488 Ly6G (clone 1A8), 127262; BV605 Ly6C (clone HK1.4), 128036; BV711 TCRβ (clone H57-597), 109243; BV605 CD4 (clone RM4-5), 100548; PE IFNγ (clone XMG1.2), 505808; and FITC CD19 (clone 6D5) 115506. From BD Pharmagen: APC IL-17A (clone TC11-18H10), 560184. BD Horizon: Fixable Viability Dye BV510 (BD 564406). For gating, see Supplementary Figs. 1 and 2.

### scRNA-seq sample preparation

Isolated cells from the colons were sorted for CD45^+^CD11b^+^CD11c^+^CD64^+^ cells to exclude neutrophils, T cells and B cells. Cells were further prepared using the 10X Single Cell comptroller per manufacturer’s instructions. scRNA-seq libraries were generated using the Chromium Next GEM Single Cell 3′ v3.1 Library & Gel Bead Kit (10X Genomics) according to the manufacturer’s protocol. In brief, emulsions were generated using the 10X Chromium Controller (10X Genomics) for a targeted recover 10,000 cells. Barcoded cDNAs were isolated from the emulsion and amplified by PCR (11 cycles). The cDNAs underwent fragmentation, end repair, and A-tailing before addition of sample indexes by PCR (16 cycles). Library products were sequenced with NovaSeq 6000.

### scRNA-seq data analysis

Cellranger v6.0.1 was used to align scRNA-seq samples to the mouse genome (mm10). Data analysis was performed using Seurat v4.3.0 R package^66^, including cell-type identification and comparative analyses between conditions. In the step of quality control, poor-quality cells with the number of expressed genes <300 or >5,000 were filtered out. We also excluded cells if their mitochondrial gene percentages were over 10%. After combining cells from all samples, we first normalized the raw count matrix and then defined the 2,000 top variable genes. We then applied PCA for dimensionality reduction and retained 30 leading principal components for cell clustering. Specifically, the shared nearest neighbour graph was constructed by calculating the Jaccard index between each cell and its 20 nearest neighbours, which was then used for cell clustering based on Louvain algorithm (with a resolution of 0.3). After identifying cluster-specific genes, we annotated cell types based on canonical marker genes (*Lyz2* for macrophages) and focused on macrophage populations in the downstream analyses. To test the differential expression of inflammatory genes between *Il10rb*-KO and *Il10rb*/*Cers2*-DKOs, we first applied ALRA^67^ to impute the gene expression profiles and then used the non-parametric Wilcoxon rank sum test to obtain their *P* values. Data are available at NCBI Gene Expression Omnibus (GEO) accession GSE252548.

### RNA-seq sample preparation

RNA was prepared with a Trizol/Qiagen RNeasy Kit hybrid protocol and submitted to Yale Center for Genome Analysis for RNA Library prep (poly A selection) and NovaSeq sequencing.

### RNA-seq data analysis

Adapter sequences were removed from the raw sequencing reads using the tool Cutadapt (https://journal.embnet.org/index.php/embnetjournal/article/view/200). STAR^68^ v2.5.3 was then used to align the trimmed sequencing reads to the mouse genome (mm10) with default parameters. After sorting the generated SAM files (as the output of alignment) with Picard Toolkit (https://broadinstitute.github.io/picard/; Broad Institute), we counted the number of reads mapped to each gene using HTSeq^69^ v0.6.1. Subsequently, we employed DESeq2^70^ v1.26.0 R package to identify significantly differential genes between naive and TLR2-activated (24 h) wild-type and *Il10*-KO BMDMs. Data are available at NCBI GEO accession GSE252547.

### Reporting summary

Further information on research design is available in the Nature Portfolio Reporting Summary linked to this article.

## Online content

Any methods, additional references, Nature Portfolio reporting summaries, source data, extended data, supplementary information, acknowledgements, peer review information; details of author contributions and competing interests; and statements of data and code availability are available at 10.1038/s41586-024-07098-5.

### Supplementary information

Supplementary Information

Reporting Summary

### Source data

Source Data Fig. 1

Source Data Fig. 2

Source Data Fig. 3

Source Data Fig. 4

Source Data Extended Data Fig. 1

Source Data Extended Data Fig. 2

Source Data Extended Data Fig. 3

Source Data Extended Data Fig. 4

Source Data Extended Data Fig. 5

Source Data Extended Data Fig. 6

Source Data Extended Data Fig. 7

Source Data Extended Data Fig. 8

Source Data Extended Data Fig. 9

## Data Availability

The datasets generated during the current study are available at NCBI GEO with accession numbers: GSE252548 (scRNA-seq) and GSE252547 (bulk RNA-seq). Source data are provided with this paper.
