# Supplementary material for: IL-10 constrains sphingolipid metabolism to limit inflammation
Source: Nature. 2024 Feb 21;627(8004):628–35. doi: 10.1038/s41586-024-07098-5 (PMC10954550; doi:10.1038/s41586-024-07098-5)
Supplement: Supplementary file 1 — Supplementary Information [file 41586_2024_7098_MOESM1_ESM.pdf]

nature portfolio

https://doi.org/10.1038/s41586-024-07098-5

Supplementary information

IL-10 constrains sphingolipid metabolism to limit inflammation

In the format provided by the authors and unedited

IL-10 constrains sphingolipid metabolism to limit inflammation

Supplemental Information Guide:

Figure 1: Example flow cytometry gating for immune cell populations purified from colons of chimeric mice.....p1

Figure 2: Example flow cytometry gating for immune cell populations purified from murine colons.....p2

Supplemental Information: **Figure 1:**

A.

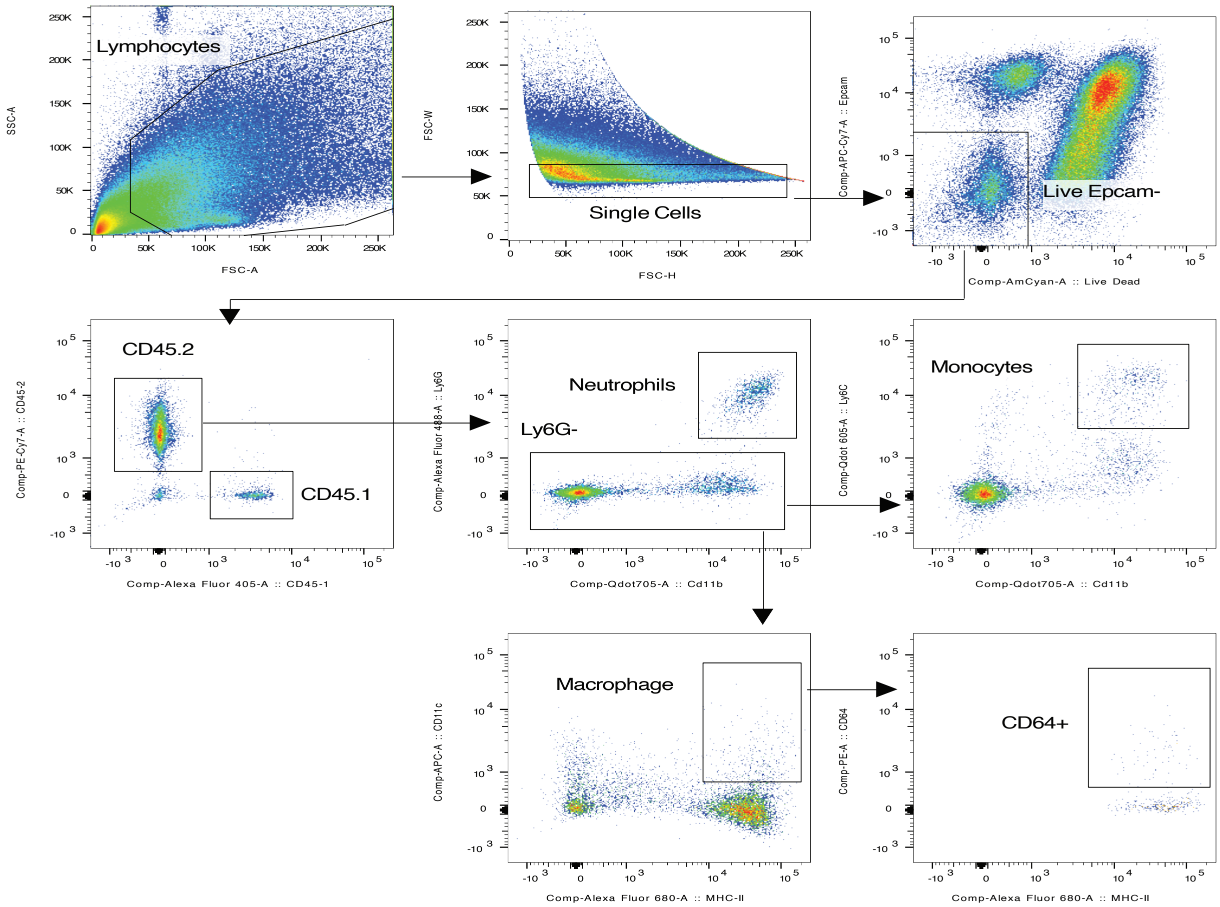

B.

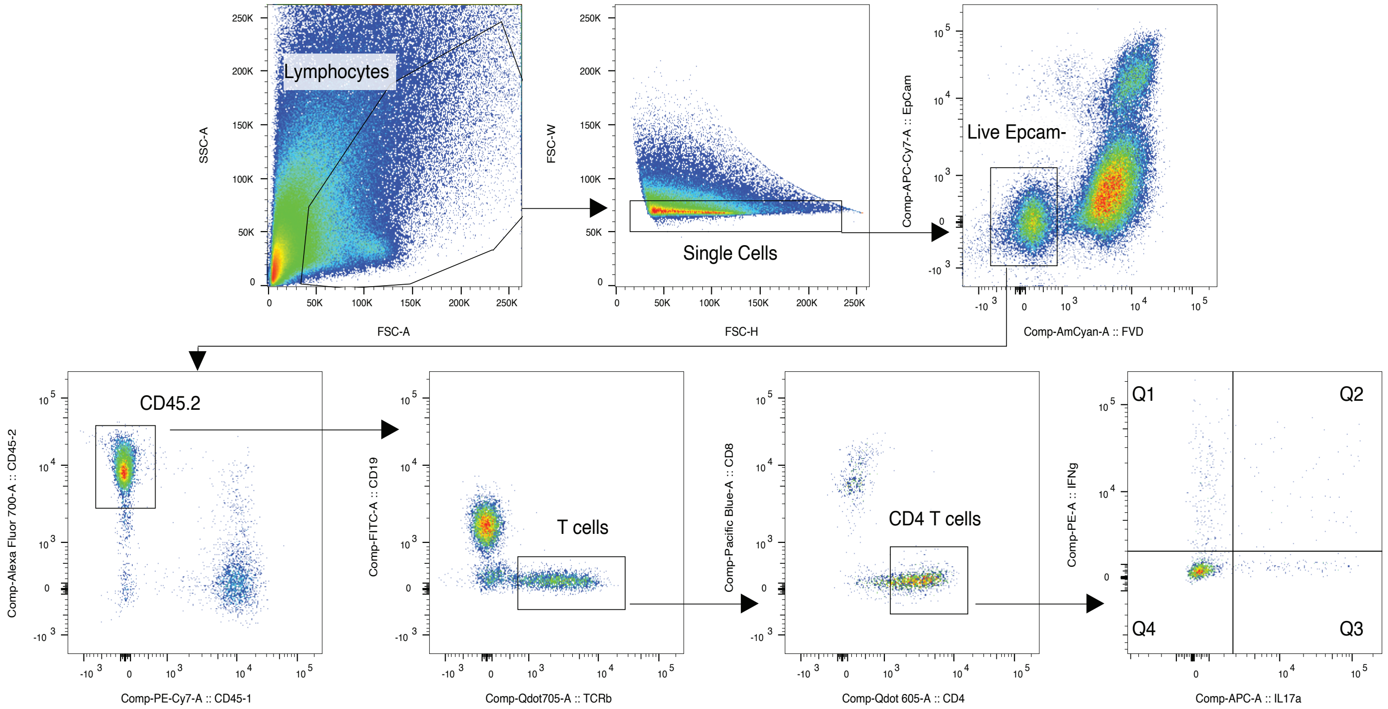

Figure 1: Example flow cytometry gates for A) myeloid and B) Lymphoid immune cell populations purified from colons of chimeric mice

Supplemental Information: **Figure 2:**

A.

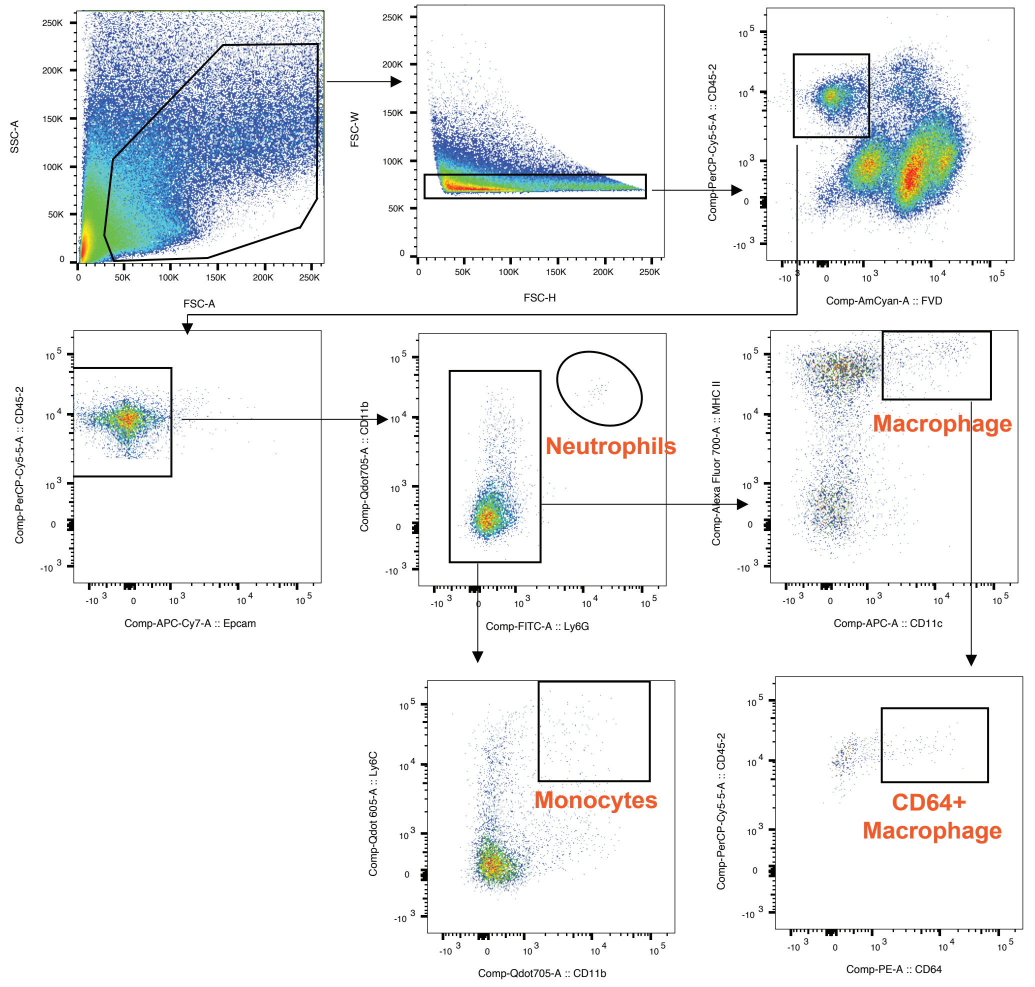

B.

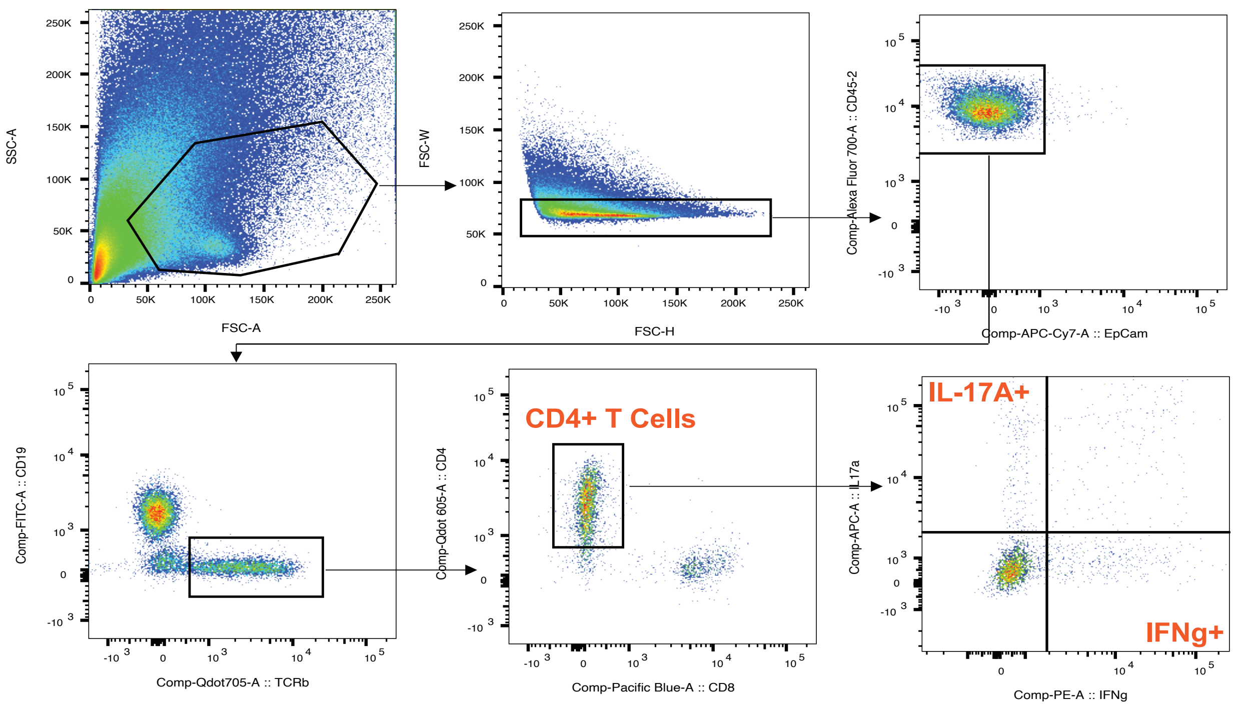

Figure 2: Example flow cytometry gates for A) myeloid and B) Lymphoid immune cell populations purified from murine colons
