# Supplementary material for: IL-10 constrains sphingolipid metabolism to limit inflammation
Source: Nature. 2024 Feb 21;627(8004):628–35. doi: 10.1038/s41586-024-07098-5 (PMC10954550; doi:10.1038/s41586-024-07098-5)
Supplement: Supplementary file 2 — Reporting Summary [file 41586_2024_7098_MOESM2_ESM.pdf]

nature portfolio

Corresponding author(s): Autumn York, Steven Bensinger, Richard Flavell

Last updated by author(s): 01/09/2024

Reporting Summary

Nature Portfolio wishes to improve the reproducibility of the work that we publish. This form provides structure for consistency and transparency in reporting. For further information on Nature Portfolio policies, see our Editorial Policies and the Editorial Policy Checklist.

Statistics

For all statistical analyses, confirm that the following items are present in the figure legend, table legend, main text, or Methods section.

n/a	Confirmed	
☐	☒	The exact sample size (n) for each experimental group/condition, given as a discrete number and unit of measurement
☐	☒	A statement on whether measurements were taken from distinct samples or whether the same sample was measured repeatedly
☐	☒	The statistical test(s) used AND whether they are one- or two-sided<br>*Only common tests should be described solely by name; describe more complex techniques in the Methods section.*
☐	☒	A description of all covariates tested
☐	☒	A description of any assumptions or corrections, such as tests of normality and adjustment for multiple comparisons
☐	☒	A full description of the statistical parameters including central tendency (e.g. means) or other basic estimates (e.g. regression coefficient) AND variation (e.g. standard deviation) or associated estimates of uncertainty (e.g. confidence intervals)
☐	☒	For null hypothesis testing, the test statistic (e.g. F, t, r) with confidence intervals, effect sizes, degrees of freedom and P value noted<br>*Give P values as exact values whenever suitable.*
☒	☐	For Bayesian analysis, information on the choice of priors and Markov chain Monte Carlo settings
☒	☐	For hierarchical and complex designs, identification of the appropriate level for tests and full reporting of outcomes
☒	☐	Estimates of effect sizes (e.g. Cohen's d, Pearson's r), indicating how they were calculated

Our web collection on statistics for biologists contains articles on many of the points above.

Software and code

Policy information about availability of computer code

Data collection	All flow cytometry data was collected with FACSDiva 7 software. Lipidomic data were collected with the Sciex Lipidyzer Platform (Sciex 5500 with DMS and Shimadzu LC-30)
Data analysis	For statistical analyses and graphs we used GraphPad Prism 9.0 and Microsoft Excel ver. 16.33 . Flow cytometry data was analyzed with FlowJo (v9 to v10.6.1). Lipidomics data analysis was performed with Lipidyzer software (v1.0). Later experiments were analyzed with the Shotgun Lipidomics Assistant (SLA v1.3).

For manuscripts utilizing custom algorithms or software that are central to the research but not yet described in published literature, software must be made available to editors and reviewers. We strongly encourage code deposition in a community repository (e.g. GitHub). See the Nature Portfolio guidelines for submitting code & software for further information.

Data

Policy information about availability of data

All manuscripts must include a data availability statement. This statement should provide the following information, where applicable:

- Accession codes, unique identifiers, or web links for publicly available datasets
- A description of any restrictions on data availability
- For clinical datasets or third party data, please ensure that the statement adheres to our policy

The datasets generated during the current study are available at NCBI GEO with accession numbers: GSE252548 (single cell) and GSE252547 (bulk RNAseq)

Human research participants

Policy information about studies involving human research participants and Sex and Gender in Research.

Reporting on sex and gender	NA
Population characteristics	NA
Recruitment	NA
Ethics oversight	NA

Note that full information on the approval of the study protocol must also be provided in the manuscript.

Field-specific reporting

Please select the one below that is the best fit for your research. If you are not sure, read the appropriate sections before making your selection.

☒ Life sciences ☐ Behavioural & social sciences ☐ Ecological, evolutionary & environmental sciences

For a reference copy of the document with all sections, see nature.com/documents/nr-reporting-summary-flat.pdf

Life sciences study design

All studies must disclose on these points even when the disclosure is negative.

Sample size	For in vivo mouse experiments, sample sizes were determined based upon the availability of mice and were the largest possible.
Data exclusions	none
Replication	All experiments were independently reproduced as stated in the figure legends.
Randomization	Animals were allocated by genotype for most in vivo experiments. There was no need for randomization in our experiments, since mice were analyzed based upon their genotype. For MUFA gavage experiments, large cohorts of either IL-10 or IL-10R KO mice were initialy screened by non-invasive (fecal lipocalin) means to determine colon inflammatory status. Animals were split into two group with equal averages of fecal lipocalin (measured by 2-tailed Students T-test) for further comparative experiments.
Blinding	Investigators were not blinded to group allocation during data collection or analysis. Blinding were not relevant to our study given that most observations made in this study were flow cytometry based and the genotypes/conditions of the samples were confirmed for multiple times prior and after the analysis.

Reporting for specific materials, systems and methods

We require information from authors about some types of materials, experimental systems and methods used in many studies. Here, indicate whether each material, system or method listed is relevant to your study. If you are not sure if a list item applies to your research, read the appropriate section before selecting a response.

Materials & experimental systems

n/a	Involved in the study	
☐	☒	Antibodies
☒	☐	Eukaryotic cell lines
☒	☐	Palaeontology and archaeology
☐	☒	Animals and other organisms
☒	☐	Clinical data
☒	☐	Dual use research of concern

Methods

n/a	Involved in the study	
☒	☐	ChIP-seq
☐	☒	Flow cytometry
☒	☐	MRI-based neuroimaging

Antibodies

Antibodies used	Flow cytometry antibodies:<br><br>Biolegend: PerCPCy5.5- Cd45.2 (Clone 104), Cat# 109828; AF700- Cd45.2 (Clone 104), Cat# 109822; BV421 CD45.1 (clone A20), cat#

	110731; BV711 Cd11b (Clone M1/70), Cat# 101242 APC CD11c (Clone N418), Cat# 117310; AF700 MHC II (I-A/I-E, clone M5/114.15.2), Cat# 107628; PE CD64 (clone X54-5/7.1), Cat# 139303; APC Cy7 Epcam (clone G8.8), Cat# 118218; AF488 Ly6G (clone 1A8), Cat# 127262; BV605 Ly6C (clone HK1.4), Cat# 128036; BV711 TCRb (clone H57-597), Cat# 109243; BV605 CD4 (clone RM4-5), Cat# 100548; PE IFNg (clone XMG1.2), Cat# 505808; FITC CD19 (clone 6D5) Cat# 115506;<br><br>BD Pharmagen: APC IL-17A (clone TC11-18H10), Cat# 560184;<br><br>BD Horizon: Fixable Viability Dye BV510 (BD #564406).<br><br>For Western blots: c-REL (Santa Cruz #SC-71); RelA (Cell Signaling #8242); TBP (Cell Signaling, clone D5C9H), Cat# 44059S; Tubulin (Cell Signaling, clone 9F3), Cat# 2128S
Validation	All antibodies are commercially available. Flow cytometry antibodies validated by manufacturer via flow cytometry. Western blot antibodies were validated by manufacturer with western blot analysis. We further validated c-Rel western blot antibody with our Rel KO animals.

Animals and other research organisms

Policy information about studies involving animals; ARRIVE guidelines recommended for reporting animal research, and Sex and Gender in Research

Laboratory animals	The following mouse stains were purchased from Jackson Labs: WT C57BL/6 (JAX 000664), IL-10 KO (JAX #002251), IL-10R KO (JAX #005027), CD45.1 PepBoy (Jax #002014) and LysM-Cre+/- (JAX #004781). CerS2 and SCD2 Flox/Flox were made in the Flavell lab and are available on request. Mice were were 8-18 weeks for experimental use. Mice were housed with 14h light/10h dark cycles in rooms maintained at 21.5 Deg C with 62% room humidity.
Wild animals	None
Reporting on sex	Groups of male or groups of female animals were used for our studies.
Field-collected samples	no field-collected animals were used in this study
Ethics oversight	All animal experimentation was performed in compliance with Yale Institutional Animal Care and Use Committee protocols.

Note that full information on the approval of the study protocol must also be provided in the manuscript.

Flow Cytometry

Plots

Confirm that:

☒ The axis labels state the marker and fluorochrome used (e.g. CD4-FITC).

☒ The axis scales are clearly visible. Include numbers along axes only for bottom left plot of group (a 'group' is an analysis of identical markers).

☒ All plots are contour plots with outliers or pseudocolor plots.

☒ A numerical value for number of cells or percentage (with statistics) is provided.

Methodology

Sample preparation	Isolation of immune cells from the colon LP: Colons were collected, flushed with 10mL PBS, and split length wise, and rinsed again in PBS. The epithelia fraction was removed with two 10mL washes of Epi Wash Buffer (1x HBSS, 5mM EDTA, 1mM DTT) at 37 deg C for 20 mins while shaking at 220RPMs. Colons were removed from Epi Wash Buffer, washed in PBS and finely minced with a razor blade. Minced tissue was transfer into 5mL Digestion buffer (1x DMEM, 5% FBS 1mg/mL Collagenase D, 0.5mg/mL DNAase) and incubated at 37 deg C for 60 mins while shaking at 220 RPMs. After digestion, samples were strained though at 70um filter and washed 2x 20mL with 1x DMEM + 5% FBS. Cells were then divided into 5 groups for different staining panels. Cells were stained with antibodies (1:400 dilution) and fixable viability dye (1:1000 dilution) at 4 deg C for 30 mins in FACS buffer (2% FBS in PBS), washed 2x, and run immediately on an LSR2 or fixed (BD Cytofix/CytoPerm #554722) for intracellular staining wit cytokine antibodies (1:100 dilution). AccuCheck cell counting beads (Invitrogen PCB100) were utilized for cell number quantification. All flow cytometry data was collected on BD LSR2s with FACSDiva 7 software. Flow cytometry data was analyzed with FlowJo (v9 to v10.6.1).
Instrument	Samples were run on BD LSR II flow cytometer, and cell sorting was performed with a BD FACSAria II sorter.
Software	All flow cytometry data was collected with FACSDiva 7 software and analyzed by FlowJo (v 9 to v10.6.1).
Cell population abundance	Abundance of cell populations were provided in each figure.

Gating strategy

Gating for Myeloid and Lymphoid cell panels is shown in Supplemental figure 3A-B and 8A-B

☒ Tick this box to confirm that a figure exemplifying the gating strategy is provided in the Supplementary Information.
